# Combined accelerated theta burst stimulation and reading instruction for the treatment of persistent developmental dyslexia: methodology and preliminary findings

**DOI:** 10.3389/fnhum.2026.1758532

**Published:** 2026-04-24

**Authors:** C. Nikki Arrington, Rachael M. Harrington, Adianes Herrera-Diaz, Kennedy Ralph, Narciso Moquete, Robin Morris

**Affiliations:** 1 Department of Psychology, Florida State University, Tallahassee, FL, United States; 2 Florida Center for Reading Research, Florida State University, Tallahassee, FL, United States; 3 Department of Psychology, Georgia State University, Atlanta, GA, United States; 4 Georgia State/Georgia Tech Center for Advanced Brain Imaging, Atlanta, GA, United States; 5 Department of Communication Sciences and Disorders, Georgia State University, Atlanta, GA, United States; 6 Neuroscience Institute, Georgia State University, Atlanta, GA, United States

**Keywords:** dyslexia, neuromodulation, reading, reading intervention, transcranial magnetic stimulation, theta burst stimulation

## Abstract

Developmental dyslexia (DD) is a neurodevelopmental disorder that results in poor reading proficiency due to atypical neurobiological attributes. Despite many opportunities to learn, the most treatment resistant cases of DD can still have severe reading problems that persist into adulthood. The current manuscript presents methodology and preliminary results for a study which aims to assess the impact of a combined accelerated theta burst stimulation (aTBS) and individualized reading instruction paradigm to improve reading outcomes in young adults with persistent DD. This multiple baseline, single-subject design assesses the effectiveness of combining aTBS and reading instruction for the treatment of DD. The current study took place over 5 cohorts with 2–4 participants per cohort. Each cohort participated in 10 nonconsecutive treatment days over five weeks, with two to three treatment days per week, each with two treatment sessions per day (20 sessions). Treatment days consisted of two sessions that included an aTBS session immediately followed by a one-on-one 30-min reading intervention administered by a trained reading specialist. Preliminary results indicate that combined aTBS and reading instruction improves oral reading fluency and accuracy above reading intervention alone. Additionally, functional neuroimaging results suggest that combined treatment has differing impact on brain regions supporting reading compared to instruction alone. The current study design provides promising preliminary support for the use of combined brain stimulation and behavioral instruction to improve reading outcomes for young adults with persistent DD.

## 1 Introduction

Developmental dyslexia (DD) in a neurodevelopmental disorder characterized by deficits in phonological processing. Neuroimaging evidence demonstrates differences in the structure and function of a left lateralized reading network in those with reading impairments such as DD (Eckert, 2004; Landi et al., 2013; Barquero et al., 2014). This network classically comprises three highly interconnected circuits, dorsal, ventral, and anterior, which support component processes of reading (Pugh et al., 2000). The dorsal (temporoparietal) circuit includes the posterior superior temporal gyrus, angular gyrus, and the supramarginal gyrus (SMG) which are primarily involved in phonological processing. The ventral (occipitotemporal) circuit is engaged in the processing of printed text and orthographic patterns while the anterior circuit, centered around inferior frontal gyrus, facilitates successfully reading comprehension through integration of text. Evidence from auditory neuroscience and the temporal sampling framework would suggest that atypical phase locking in this network, particularly with respect Delta and Theta bands, contribute to phonological deficits that are hallmark in DD (Goswami, 2011). More recently, the neural noise hypothesis of DD suggests that an excitation/inhibition imbalance within these circuits disrupts the multisensory integration that is necessary for proper print-speech mapping involved in learning to read (Hancock et al., 2017).

Despite many opportunities to learn, frequently using quality educational approaches and well validated treatments, the most persistent cases of DD can still have severe reading problems that persist into adulthood (Shaywitz and Shaywitz, 2005; Fletcher, 2009). Adults with persistent DD represent the most heterogenous portion of the DD population with deficits beyond that of phonological processing observed in children with DD (Gregg et al., 2006; Swanson, 2012). This can often make it more difficult to identify targeted treatment options. High rates of co-morbid disorders such as depression/anxiety and ADHD are often observed, compounding difficulties with treatment outcomes (Miranda et al., 2011; Hendren et al., 2018; Moll et al., 2020). Additionally, this population may not be responsive to traditional, educationally-based treatments as the neural circuits that underpin and influence reading development have less potential for neuroplastic change as one ages (Kızılaslan and Tuncay, 2023). Although there are several cognitive and behavioral remediation techniques that can be effective for many children with DD (Swanson, 1999), unfortunately, there are few additional treatment options for adults with persistent, treatment-resistant DD (Greenberg et al., 2011).

While adults with DD demonstrate similar reading and behavioral deficits as those of younger children with DD, neuroimaging data suggest larger and differing areas of atypical activation in the adult brain’s reading network (Richlan et al., 2011). These differences may suggest a neural basis for the more intractable and neurogenetically based cases of DD that are particularly resistant to traditional treatments. Adults with DD therefore provide a window into critical neurobiological and neurocognitive factors that distinguish them from other cases of remediated DD. In addition, due to their age-related decreased potential for neuroplasticity in those neural circuits that support reading, making changes in their reading behaviors is particularly challenging. Inducing neuroplasticity with noninvasive brain stimulation prior to reading instruction may facilitate learning for those with persistent DD. As such, this population is ideally suited for evaluating a neuromodulatory adjunct to treatment.

Repetitive transcranial magnetic stimulation (rTMS) has been shown to successfully treat clinical disorders and is a noninvasive means for inducing neuroplastic change (Hallett, 2007). A recent review of the literature has shown that rTMS can modulate reading processes in both typical and impaired readers (Arrington et al., 2023). More specifically, stimulation of a node within the dorsal reading circuit was shown to modulate phonological and semantic processing. Intermittent theta burst stimulation (TBS), a type of rTMS, delivered to the left SMG has been shown to selectively improve phonological processing while also increasing activation in reading circuit related brain regions (Harrington et al., 2023; Herrera-Diaz et al., 2025). Research suggests that non-invasive brain stimulation methods, such as TBS, have the potential to provide the foundation for developing a neuromodulation-based treatment model for persistent DD (Costanzo et al., 2013; Rezaei et al., 2025). Non-invasive brain stimulation may serve to help normalize or stabilize neural noise that interferes with learning to read (Hancock et al., 2017). More specifically, TBS may help to modulate atypical low-frequency phase locking mechanisms that negatively impact phonological processing in DD (Goswami, 2011). It has been shown that behavioral and cognitive treatments can have improved outcomes when paired with neuromodulation techniques (Hara et al., 2016; Tsagaris et al., 2016; Nissim et al., 2020). With TBS it is possible to change cortical excitability in the targeted neural network for up to 60 min (Harrington et al., 2023; Huang et al., 2005), during which time more focused behavioral remediation can occur. During this time of induced cortical excitability, the neural networks supporting cognitive functions, such as reading, may be more sensitive to targeted behavioral remediation techniques. Applying this principle by combining TBS and behavioral remediation has been shown to safely and effectively treat neuropsychiatric disorders such as depression, obsessive-compulsive disorder, and schizophrenia (Rachid, 2017), as well as reducing symptoms of addictive behaviors such as those seen with substance abuse and compulsive gambling (Rachid, 2017; Zhao et al., 2020). Additionally, combined TBS approaches have been shown to improve cognitive functioning such as working memory and executive function (Wu et al., 2020).

Traditional rTMS treatment protocols typically require stimulation sessions lasting up to 20 min per day, over the course of several weeks. An accelerated treatment paradigm which utilizes multiple stimulation sessions in a single day, has been shown to significantly reduce treatment time while improving behavioral outcomes, with no increased safety risks (Chen et al., 2020; Kaster et al., 2020; Wu et al., 2020). Accelerated treatment using TBS is reported to produce the enhanced benefits of a typical high frequency rTMS treatment and is equally well tolerated (Blumberger et al., 2021; Wu et al., 2022; Yu et al., 2020). In fact, accelerated TBS (aTBS) has been shown to produce significant improvements in cognitive performance above that of other accelerated rTMS paradigms in both neurotypical and impaired populations (Wu et al., 2020, 2021; Peng et al., 2025). These improvements are even further enhanced when aTBS is combined with a behavioral or cognitive intervention (Bariani et al., 2026; Luo et al., 2026). Behavioral effects of a single multi-session TBS treatment day have also been shown to last up to 32 h post stimulation (Nyffeler et al., 2009). In addition to the superior cognitive outcomes associated with such aTBS protocols, it also has increased feasibility, with each stimulation period lasting approximately 3 min as compared to the typical 20 min (Tao et al., 2025). Combined, these benefits of an accelerated stimulation treatment paradigm may serve as an ideal adjunct to educational/behavioral reading interventions for DD.

The current manuscript aims to present the methodology and feasibility of a combined aTBS protocol and educational intervention paradigm for the treatment of specific fluency and accuracy reading difficulties in young adults with DD. Specifically, we utilized aTBS as a tool to modulate cortical excitability prior to explicit reading instruction to enhance specific reading outcomes in this population. The current manuscript follows the single-case reporting guidelines in behavioral interventions (SCRIBE; Tate et al., 2016) for reporting evidence for the feasibility and tolerance of combining aTBS and individualized educational intervention for the treatment of persistent DD.

## 2 Methods

### 2.1 Study design

The current study used a single-subject, multiple baseline design with *a priori* phase sequence to account for intervention effects which were expected to have non-reversible, carry forward effects (Barger-Anderson et al., 2004). A cohort design was used with cohort group participants involved in concurrent study sessions. Study involvement consisted of three stages: (1) screening and pre-treatment, (2) accelerated treatment, and (3) post-treatment with optional longitudinal follow-up.

#### 2.1.1 Screening and pre-treatment stage

All participants completed a battery of standardized language, reading, and cognitive assessments for screening and inclusion in the treatment program. Those participants that qualified for the accelerated treatment stage (see Selection Criteria and Participant Characteristics) completed additional pre-treatment standardized and experimental measures (see Measures and materials section). Participants also completed a pre-treatment MRI scan consisting of a series of structural, functional, and neurochemical sequences designed to quantify pre-treatment characteristics of the reading network.

#### 2.1.2 Accelerated treatment stage

**Figure 1:**
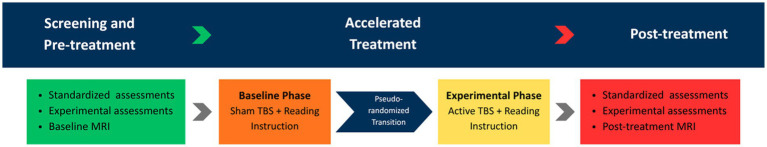
Graphical illustration of study design.

The accelerated treatment stage consisted of a baseline and experimental phase with a total of 10 nonconsecutive treatment days (Figure 1). Treatment days took place over five weeks with two to three treatment days per week, each with two treatment sessions per day (20 sessions total). A single treatment day consisted of approximately six hours of study participation. Each treatment day consisted of participants completing a morning and afternoon session with a minimum of two hours (mean = 2.34 h) between each session. The baseline phase consisted of a minimum of two days (4 sessions) of combined sham TBS and targeted reading instruction. The experimental phase consisted of a minimum of five days (10 sessions) of combined active TBS and reading instruction. Experimental phase days followed the same protocol and schedule as the baseline phase, with only type of stimulation differing. All participants within a single cohort group began the baseline phase on the same day. Transition from baseline to experimental phase was pseudorandomized to ensure an equal number of experimental sessions across cohorts and SRA reader levels and varied amongst participants in a single cohort.

**Figure 2:**
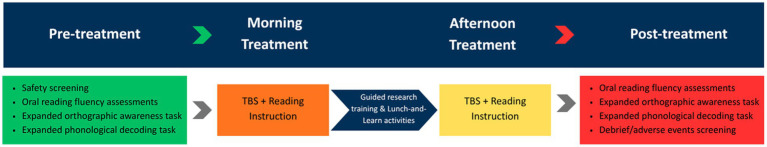
Graphical illustration of single day of accelerated treatment, inclusive of morning and afternoon sessions.

Each treatment day consisted of: (1) pre-treatment safety screening, (2) pre-treatment assessments, (3) morning treatment session, (4) guided research training and lunch-and-learn activities, (5) afternoon treatment session, and (6) post-treatment assessments and daily debrief (Figure 2). Pre-treatment safety screenings of TBS contraindications were completed each morning by qualified study staff. After screening, participants completed daily pre-treatment assessments. Treatment sessions, both morning and afternoon, contained TBS (either active or sham) immediately followed by 30 min of one-on-one targeted reading instruction with an experienced and trained program reading specialist instructor. An individual combined TBS/reading instruction session lasted a total of one hour. Between morning and afternoon treatment sessions, participants engaged in participatory research training activities. These activities included guided research education such as research ethics, data analysis techniques, and an introduction to neuroimaging. Participants also engaged in lunch-and-learn group discussions as part of their research training, covering topics such as the neurobiology of reading and the use of neuromodulation in language research. Treatment days concluded with post-treatment assessments, consistent with those administered pre-treatment. A daily debrief and screening for adverse events was also completed at the conclusion of each treatment day.

#### 2.1.3 Post-treatment stage

After completion of the accelerated treatment stage (mean = 2.23 days post treatment), each participant completed a series of experimental and standardized assessments, consistent with those completed at pre-treatment. They also completed a post-treatment MRI scan consisting of sequences consistent with the pre-treatment scan, designed to quantify treatment effects on the reading network. Those participants who consented to the longitudinal follow-up portion of the study also completed additional assessments and an MRI scan six to nine months (mean = 250 days) post treatment.

#### 2.1.4 Procedural changes

In the event that a participant was no longer eligible to receive TBS (i.e., change in health status) or they withdrew from the TBS portion of the study, participants were eligible to continue participation in the reading treatment alone. A transition to reading treatment only did not impact overall study completion. This was the case for two participants. In one case, after successfully completing 8 sessions (4 days) of sham stimulation, one participant reported specific discomfort during the first session of active TBS. To maintain comfort and safety, this participants was switched to a sham only protocol, without their knowledge, with ongoing reading instruction and was able to continue study involvement. One participant received a combined 15 baseline and experimental sessions and then completed five instruction-only sessions after reporting discomfort associated with active stimulation.

One participant was ineligible to receive TBS due to a contraindication identified after the baseline MRI and was thus assigned to reading instruction only prior to beginning treatment. On one occasion a participant completed their morning pre-treatment assessments and treatment session but was unable to complete the afternoon treatment and post-treatment assessments. This half day was made up on a later date at the end of study participation to allow for study completion. In only one case did a participant not complete all study sessions as the participant was excluded from the study due to excessive absenteeism after completing four treatment days (two baseline and two experimental).

#### 2.1.5 Replication

The current study consisted of a total of 14 participants across five cohorts. Each cohort consisted of two to four participants. Study design was replicated concurrently within a cohort.

#### 2.1.6 Blinding

All participants were blinded to treatment condition as well as date of transition from baseline (sham stimulation) to experimental (active stimulation) phase. Research personnel administering targeted reading instruction were also blind to TBS condition. Daily assessments of target reading outcomes were administered by research personnel external to the TBS and reading instruction personnel and blinded to date of transition from baseline to experimental phase.

### 2.2 Participants

#### 2.2.1 Selection criteria and participant characteristics

Native English speakers between 18 and 30 years of age (mean = 21.86 years) with a documented or suspected history of DD or other developmental reading disability were recruited for the study. Individuals were included if they met study designated criteria for PDD, identified as scoring below age-norm expectations (standard score <85, <15%ile for age) on at least one subtest of study administered standardized reading assessments (TOWRE-2, WJ-IV Broad Score, GORT) and tested at a reading error and reduced fluency level that placed them into the study reading intervention curriculum (level B2 or C on the *SRA Corrective Reading Decoding Placement Test;* see Table 1). These reading criteria were designed to identify adult participants with significantly below average reading accuracy and/or fluency abilities (Gregg et al., 2006; Swanson, 2012). Included participants were also required to have at least a low average or higher functioning as indicated by the Wechsler Abbreviated Scale of Intelligence (WASI-2, Wechsler, 2011) *Vocabulary* or *Matrix Reasoning* subtest (*T* score > = 40). To best represent the heterogeneity of the adult DD population, qualifying participants with commonly occurring co-morbid disorders (i.e., ADHD, anxiety/depression) were included if the co-morbid disorder was currently well controlled and any medication being taken was not considered a TBS contraindication. Individuals with significant hearing or vision impairments that inhibited study involvement or those with MRI or TBS contraindications (i.e., metal in body or certain health conditions) were excluded from the study. Those who were unable to attend all required in-person study dates were also excluded.

**Table 1:** Summary of participant demographics and characteristics.

Case number	Age/gender	Ethnicity	Previous intervention	Years of education	WASI FSIQ-2	WJ-IV basic CST	TOWRE form A CST	GORT accuracy SS	SRA level
Case 1	22/NB	Caucasian	NR	16	99	93^A^	95	9	C
Case 2	21/F	Asian American	Yes	15	102	90	68	3	B2
Case 3	20/F	Caucasian	Yes	15	111	98 ^A^	102	9	C
Case 4	19/M	African American	No	13	99	78	81	4	B2
Case 5	21/F	Caucasian	Yes	14	125	75	72	1	B2
Case 6	25/NB	African American	NR	16	99	104	76	11	C
Case 7	20/F	Caucasian	Yes	14	97	90 ^A^	89	7	C
Case 8	21/F	Caucasian	Yes	14	109	83	78	6	C
Case 9	28/F	Caucasian	NR	NR	93	93	78	5	B2
Case 10	23/F	Caucasian	No	13	95	74	72	3	C
Case 11	19/F	African American	NR	14	86	80	86	4	C
Case 12	19/F	Hispanic	No	13	78 ^B^	86 ^A^	96	5	C
Case 13	26/F	African American	Yes	12	90	105	82	6	C
Case 14	22/F	African American	NR	NR	80	97	83	9	B2

CST, composite standard score; F, female; GORT, gray oral reading test; M, male; NR, no response; NB, non-binary; SS, scaled score; TOWRE, test of word reading efficiency; WASI, Wechsler Abbreviated Scale of Intelligence; WJ-IV, Woodcock-Johnson IV tests of achievement.

^A^Inclusion criteria met with a standard score below 85 on one of the following subtests of the WJ-IV, word attack, oral reading fluency, passage comprehension.

^B^Inclusion criteria met with a *T* score equal to 40 on WASI matrix reasoning.

### 2.3 Context

All in-person study components were conducted at the Georgia State University/Georgia Institute of Technology Center for Advanced Brain Imaging (CABI) in Atlanta, Georgia. CABI is a research-dedicated facility which provides access to resources necessary to facilitate study activities. This includes private testing rooms for behavioral assessments and educational intervention as well as a large meeting space for lunch-and-learn and research training activities. All portions of the study protocol were administered by an appropriately trained member of the study staff. TBS was administered in a quiet room with the participant seated comfortably in an appropriate neuromodulation chair. At least two members of the study team were present during TBS administration, including a safety monitor to assess adverse reactions. A staff nurse was available during TBS sessions to ensure safety. A review of TBS contraindications and adverse events was also completed at the end of each treatment day.

### 2.4 Approvals

This study was reviewed and approved by the Georgia State University Institutional Review Board (protocol H22625). Written informed consent was provided via electronic signature or in-person upon their initial screening visit. A copy of the consent form was emailed in advance for the participant to review prior to signing. Study staff were available to review and discuss the consent, and read it along with the participant, as needed. An audio version of each consent form was also available on the study website (https://sites.gsu.edu/rare-internship/audio-consent-forms/).

Participants provided written informed consent at three stages of study involvement: (1) language, reading, and cognition battery screening (i.e., pre-screening), (2) intensive program, which consisted of baseline behavioral assessments and MRI, accelerated treatment phase, and post-treatment assessments and MRI, and (3) longitudinal follow-up. All potential participants (*n* = 39) provided consent into the initial screening phase prior to study involvement. Those that met criteria for inclusion in the treatment program (standard score <85 on at least one reading subtest and level B2 or C on the *SRA Corrective Reading Decoding Placement Test*) were consented into the intensive treatment program (*n* = 17). Three potential participants that consented to the intensive treatment program were unable to participate due to scheduling conflicts. All participants who completed the accelerated treatment stage were provided with the option of involvement in the longitudinal follow up portion of the study (consented = 11).

## 3 Materials and equipment

### 3.1 Screening and pre-treatment stage

#### 3.1.1 Reading

The *Gray Oral Reading Test* – 5 (GORT-5) (Wiederholt and Bryant, 2012) is an age-normed assessment designed to assess oral reading abilities. The GORT contains 16 developmentally sequenced reading passages with five relevant comprehension questions for each passage with high content and construct validity (0.33 to 0.77) (Hall and Tannebaum, 2013). The oral reading index produces a standard score based on age-matched norms as a composite of reading accuracy, fluency, and comprehension which was used as a measure of generalized reading ability.

The orthographic awareness (OA) task is a computerized forced-choice, reaction time assessment which measures how quickly and automatically participants can identify valid orthographic patterns. This well-validated and well-replicated experimental task (Olson et al., 1994) is used to measure the speed at which participants can identify whether or not a string of letters represents a correctly spelled real word. Participants see a letter string on the screen that either represents a correctly spelled real word (e.g., “flute”) or an incorrectly spelled word (e.g., “fone”). They are asked to judge via button press whether the letter string is a “real word” or “not a real word.” Correctly and incorrectly spelled words are intermixed pseudo-randomly with response times and accuracy rates recorded for a total of 32 trials. The median response time across correct trials was calculated for each participant.

The phonological decoding (PD) task is a computerized forced-choice, reaction time assessment of phonological decoding skills which measures the speed and accuracy of participants’ ability to phonetically decode a string of letters. This task is simple with accuracy above the level of chance and has proven highly sensitive to individual differences in reading skills (Olson et al., 1994). Participants see a letter string on the screen and are asked to judge via button press whether the letter string can be pronounced like a real word (e.g., “roze”) or not (e.g., “heaf”). Pronounceable and unpronounceable pseudowords are intermixed pseudo-randomly with response times and accuracy rates recorded for a total of 50 trials. The median response time across correct trials is calculated for each participant.

The SRA *Corrective Reading* Decoding Placement Test (Engelmann, 1988) is a criterion-referenced assessment for decoding and reading accuracy skills and oral reading fluency. Participants are required to read aloud four progressively more difficult passages as quickly and accurately as possible. Scores are based on total number of decoding errors and the amount of time required to read each passage. This measure was used to establish treatment program placement and baseline instructional starting point.

The *Test of Word Reading Efficiency* – Second Edition (TOWRE-2; Torgesen et al., 2012) is a speeded assessment for word reading efficiency. TOWRE-2 requires reading of sight words (Sight Word Efficiency) and pseudowords (Phonemic Decoding Efficiency). On both subtests, the items are ordered from easiest to most difficult, with a standardized score produced based on number of items read correctly in 45 s. Each subtest of the TOWRE-2 offers four alternate forms (A–D) with the average reliability coefficients for the subtests exceeding 0.90. The average test–retest coefficients for different forms of the subtests are 0.87. Form A for each subtest was administered during the pre-treatment stage.

Subtests of the *Woodcock-Johnson IV Tests of Achievement* (*WJ-IV*; Schrank et al., 2014) which make up the standardized Broad and Basic Reading scores were used to index generalized reading ability. Each of these subscales are age-normed and have been shown to have high clinical validity in reading impaired populations (Villarreal, 2015). The Letter-Word Identification subtest requires accurate pronunciation of single words by reading aloud from an increasingly more difficult list of sight words. Word Attack measures the ability to apply phonics and structural analysis skills to the pronunciation of unfamiliar non-words by reading an increasingly more difficult list of pseudowords. The Passage Comprehension subtest measures the ability to understand written discourse. It requires the silent reading of short passages with increasing difficulty Participants must identify a missing key word that makes sense in the context of the passage. Oral Reading measures oral sentence reading fluency through the reading of progressively more complex paragraphs. Performance is evaluated based on errors in mispronunciation, omission, insertion, substitution, hesitation, repetition, transposition, and/or ignoring punctuation. The Sentence Reading Fluency subtest assesses the ability to quickly and silently read and comprehend a list of increasingly more complex sentences. Participants are instructed to complete as many items as possible in a 3-min time limit by indicating whether each sentence is true or false.

#### 3.1.2 Language

The Comprehensive Test of Phonological Processing – 2 (CTOPP-2) Wagner et al. (2013) is a well-validated, standardized assessment of phonological processing abilities, normed for ages 4–24 with a range of reading abilities as a prerequisite to reading fluency. Collected subtests included Blending Words and Elision. The Blending Words subtest assesses the ability to blend individual morphemes and phonemes into whole words. The Elision subtest assesses the ability to parse morphemes and phonemes from whole words. These subtests have been shown to have high reliability and validity (0.69 to 0.83) in adults with DD (Nanda et al., 2014).

Peabody Picture Vocabulary Test Fifth Edition (PPVT-5; Dunn, 2019) is a norm-referenced (2:6–90 + Years) measure of receptive vocabulary based on words in the Standard American English. It measures receptive vocabulary acquisition and can contribute useful information when assessing receptive vocabulary, as part of a language evaluation, across the lifespan. Participants choose the picture that best represents the word spoken by the examiner from a field of four pictures.

#### 3.1.3 Cognition

*Wechsler Abbreviated Scale of Intelligence* - II (WASI-II; Wechsler, 2011) is a measure of general verbal and non-verbal cognitive abilities, as well as an abbreviated index of FSIQ, in individuals aged 6 to 90 years. The Vocabulary subtest is an index of vocabulary and language function and is assessed by asking participants to provide definitions of concepts. The Matrix Reasoning subtest is an index of non-verbal visuospatial problem solving. Together, these subtests provide an index of FSIQ-2 with high reliability and validity (0.91 to 0.99) in typically developing and clinical populations (Irby and Floyd, 2013).

Adaptive Cognitive Evaluation Explorer (ACE-X) is a mobile, computerized cognitive assessment battery delivered via iPad, designed to replicate validated gold-standard tasks that assess different aspects of executive function (O’Laughlin et al., 2025). The Response Time subtest was used to index basic response time via finger tap response for a colored stimuli. The Boxed subtest was used to assess visual search interference processing. In this task, participants are instructed to identify a target item while ignoring distractors. Color Tricker is based on the Stroop task (Logan et al., 1984) and is designed to measure response inhibition performance. Selective attention was assessed using the Compass subtest which is based on the Posner cueing paradigm (Posner and Cohen, 1984). Gem Chaser and Backwards Gem Chaser subtests are consistent with the paradigm set forth in the Corsi Block Task (Forward and Reverse) in order to measure visuospatial span and working memory capacity, respectively (Berch et al., 1998).

The Math Fluency age-normed subtest of the *WJ-IV* (Schrank et al., 2014) was used to index non-treatment targeted academic cognitive abilities. This subtest measures the ability to quickly solve simple addition, subtraction, division, and multiplication problems within a 3-min time limit.

#### 3.1.4 MRI scan sequences

Images were acquired using a 3 T Siemens PRISMA Fit scanner located at the GSU/GTech Center for Advanced Brain Imaging (Atlanta). Data acquisition and scan parameters were kept consistent across participants.

##### 3.1.4.1 Anatomical imaging

High-resolution, three-dimensional 1.0×1.0×1.0 mm T1-weighted sagittal acquisition was acquired to characterize anatomical properties of the reading network (MPRAGE multi-shell pulse sequence; TE1 = 1.69 ms, TE2 = 3.55 ms, TE3 = 5.41 ms, TE4 = 7.27 ms; TR = 2,530 ms; FA = 7 degrees; FOV = 256×256 pixels; slice thickness 1.0 mm; 256 slices). T2*-weighted images were collected in the same orientation (matrix size = 32 × 32; voxel size = 1.0×1.0×1.0 mm; FoV = 256 mm; TR = 3,390 ms; TE = 388 ms).

##### 3.1.4.2 Diffusion-weighted imaging

Diffusion-weighted images were acquired to characterize properties of white matter diffusivity (FoV = 220x220mm^2^, matrix = 140×140, 60 transverse slices, 2 mm isotropic resolution, anterior-to-posterior (AP) phase encoding, TR = 2,830 ms, TE = 83 ms, FA = 78°, 3 simultaneous slices, acquisition BW = 1,516 Hz/pixel, 9 b = 0 s/mm^2^ images, 140 directions at b = 2,000 s/mm^2^). A posterior-to-anterior (PA) phase encoding b = 0 s/mm^2^ volume was collected to correct for EPI-induced distortions during processing.

##### 3.1.4.3 Functional imaging

Functional MRI sequences were collected for two different reading/language based functional tasks, as well as resting state functional MRI.

The Fast fMRI Localizer (Fast-Loc) task is a passive reading task that is highly effective at identifying brain regions that are sensitive to domain-specific linguistic properties (Malins et al., 2016; Arrington et al., 2019). Participants completed three runs of this task, each of which consisted of 158 volumes, lasting 5:22 min (TR = 2000 ms, TE = 30 ms, flip angle = 90°, FOV = 220 mm, voxel size = 3.4×3.4×4.0 mm, with 32 slices acquired in transversal orientation and phase encoding direction A/P). On each trial, four items were presented in visual modality in rapid succession. Participants were asked to respond via button press on those trials in which the third and fourth items of the set were identical (i.e., oddball trials). Oddball trials occurred in one third of the trials in each condition. Standard trials were defined as trials in which all four stimuli in the set differed and therefore, did not require a response. Each run contained 48 trials that were evenly distributed across six visual stimulus conditions (for a total of 24 trials for each condition across the whole experiment). Across all trials, the time between trial onset was jittered between 4 and 13 s. Trials started either at the beginning or the middle of a TR, with the likelihood for this equally balanced across all stimulus conditions (Belin et al., 1999).

The Picture Identification (PID) task was collected in cohorts two through five. In this task a cue-target identity matching functional task used to characterize the neural circuitry for print and speech (Preston et al., 2010; Malins et al., 2018). The PID task is simple with high accuracy, has been used successfully with this age group, and has proven highly sensitive to individual differences in reading skills as well as to network changes in response to TBS (Herrera-Diaz et al., 2025). Participants completed two runs of this task, each of which consisted of 113 volumes, lasting 3:52 min (TR = 2000 ms, TE = 30 ms, flip angle = 90°, FOV = 220 mm, voxel size = 3.4×3.4×4.0 mm, with 32 slices acquired in transversal orientation and phase encoding direction A/P). In this task, participants see a picture cue and are then presented with potential “targets” (i.e., written or spoken word) and asked to judge with a button press if the target stimulus matches the picture cue. Matching trials and mismatching trials consisted of 1/6 and 5/6, respectively, of all trials presented. Picture cues remained on the screen for 40–65 s, before being replaced by a new picture. The target items were presented following an event-related protocol with written words displayed below the picture (for 3,000 ms) or auditory words delivered via headphones. Across all trials, the time between trial onset was jittered between 4 and 13 s.

Participants also completed an open eye resting state functional sequence lasting 7:38 min. Resting state data will be collected using a conventional BOLD EPI sequence (TR = 750 ms, TE = 32 ms, flip angle = 52°, FOV = 220 mm, voxel size = 2.5×2.5×2.5 mm, with 50 slices acquired in transversal orientation and phase encoding direction A/P). During this time a white fixation cross was presented on the middle of a black screen. Participants were instructed to focus on the image while letting their mind relax, thinking about nothing in particular.

##### 3.1.4.4 Magnetic resonance spectroscopy

The J-edited (Rothman et al., 1993) Magnetic resonance spectroscopy (MRS) acquisition utilized the Center for Magnetic Resonance Research (CMRR) Spectroscopy Tools Mescher-Garwood Point Resolved Spectroscopy (MEGA-PRESS) (Mescher et al., 1998) sequence to separate the small GABA+ signals from the rest of the MR spectrum (TR = 2000 ms, TE = 68 ms, voxel size = 3x3x3 cm^3^ = 27 mL, acquisition bandwidth = 2000 Hz, vector size = 1,024, VAPOR water suppression bandwidth = 135 Hz, editing pulse bandwidth = 53 Hz, ON editing pulse = 1.9 ppm, OFF editing pulse = 7.5 ppm, 87 pairs of ON/OFF acquisitions, total scan duration = 6 min). The CMRR Spectroscopy Tools FAST(EST)MAP (Gruetter, 1993; Gruetter and Tkáč, 2000) was used to achieve a high-quality shim. Each free induction decay (FID) was collected and stored separately for use in preprocessing. The MRS voxel was planned over the left hemisphere inferior frontal region (anterior portion of the ascending ramus of the Sylvian fissure) using a high-resolution multi-echo T1w by a trained technician. An unsuppressed water (H_2_O) spectrum with matching acquisition parameters was also collected from the same region, except that the TR = 10 s to allow for full T_1_ relaxation.

#### 3.1.5 Accelerated treatment stage

Participants completed a short battery of reading assessments, taking approximately 15 min, at the beginning and end of each treatment day (pre-treatment/post-treatment). These assessments were designed to assess skills directly targeted by the reading instruction, as well as component reading processes related to fluency outcomes but not directly targeted.

Daily oral reading fluency (ORF) abilities were assessed using a selection of passages from the Texas Middle School Fluency Assessment (TMSFA). The TMSFA is a reading instrument designed to measure growth in reading and has been shown reliable for the ongoing evaluation of reading skills (Fletcher, 2009; Santi et al., 2016). Although originally presented for assessment of middle school reading fluency and comprehension skills, previous research and passage difficulty, as indicated by Lexile level (Smith et al., 2016), indicate an appropriateness of the measure for readers within the range of those in the current study (Santi et al., 2016).

The passage reading fluency measure consisted of a set of four passages, one each day, for a total of 40 passages. Passages were both narrative and expository, with length of passages from approximately 350 to 700 words each. Passages were grouped by difficulty based on Lexile level, ranging in difficulty from 1,020 to 1,460 Lexile (Stenner, 2007). Passages at or below 1,230 Lexiles (mean = 1120.5) were identified as on reading level whereas passages at or above 1,250 Lexiles (mean = 1338.5) were identified as above reading level. Each set of passages consisted of two on-level and two above-level passages. Participants read two passages from a set, one on-level and one-above level, pre-treatment and post-treatment each day. Passage sets were randomized across participants and treatment days. Participants were instructed to read each passage as quickly and accurately as possible for three minutes. They then answered five explicit and inferential questions about the story. ORF scores were recorded individually as the average number of words read correctly per minute of reading (WCPM) and number of correctly answered story questions.

Expanded PD and OA computerized tasks were developed in-house, consistent with those completed at baseline, for use pre- and post-treatment days. Items for the PD task lists were gathered from the ARC nonword database (Rastle et al., 2002). A list of 500 4–5 letter pseudohomophones and 4–5 letter nonwords with only legal bigrams were generated. Items for the OA task were generated using the English Lexicon Project (Balota et al., 2007). A list of 500 4–5 letter words and nonwords were generated. All real words had a log-transformed frequency from the Hyperspace Analog to Language (HAL; Lund and Burgess, 1996) corpus greater than 5 and all words were restrained between 1–2 syllables. Lists were confirmed to not contain any duplicates of nonwords between or within the two tasks. Duplicates of the real word associated with each pseudohomophone and generated real words were also confirmed to not be shared between the two tasks. Real words associated with each pseudohomophone were confirmed to have log-transformed frequency greater than 5. This list was then screened by study staff to remove any slang or inappropriate words or words that may fall outside of their assigned list in modern context (e.g., yeet). Master lists were then randomly sorted and assigned to 10 lists per task. Each list contained 80 items (40 real words and 40 nonwords for the OA task and 40 pseudohomophones and 40 nonpseudohomophones for the PD task). Lists were balanced based on exported database characteristics, including length, frequency, initial and final phonemes, and neighborhood characteristics. Lists were programmed in E-Prime 3.0, and order of list administration was randomized across participants and treatment days.

#### 3.1.6 Post-treatment stage

##### 3.1.6.1 Behavioral assessments

A battery of assessments, consisting of a subset of those done at screening/pre-treatment were completed at the conclusion of the accelerated treatment stage. Standardized reading and language assessments included TOWRE-2 Form B, CTOPP-2 Blending Words and Elison subtests, as well as the Reading Fluency and Math Fluency subtests of the WJ-IV. A secondary list of the Olson et al. (1994) based PD and OA computerized tasks were also administered post treatment. The Response Time, Boxed, Color Tricker, Compass, Gem Chaser, and Backwards Gem Chaser subtests of ACE-X were collected to index post treatment executive functioning.

##### 3.1.6.2 MRI scan sequences

Post-treatment MRI scans, consistent with those collected pretreatment, were collected during the post-treatment stage to quantify reading network changes following the accelerated treatment program. Data acquisition and scan parameters were kept consistent across participants and time points.

### 3.2 Intervention

#### 3.2.1 Accelerated theta burst stimulation

Participants received two stimulation sessions per day in which either active or sham intermittent TBS was delivered to the left supramarginal gyrus (SMG) following parameters established by Huang et al. (2005). Stimulation sessions were administered by a trained TMS technician and overseen by the study nurse. During stimulation participants were seated in a comfortable chair with limbs uncrossed in a comfortable position. A cap was placed over their head to protect from abrasion and ease target site localization between sessions. Each session began by locating the hand area of the motor cortex, anatomical landmarks between the nasion and inion, and one-third of the distance between the vertex and the tragus of the left ear were indicated on the cap. The primary motor cortex was pinpointed by applying single pulses of TMS using a figure-of-eight coil (C-B60) connected to a MagVenture MagPro X100 stimulator (MagVenture, Lucernemarken 15 DK-3520 Farnum, Sjaelland, Denmark). Muscle contraction of the right thumb and/or index finger was visually monitored to confirm correct placement. Once the motor hotspot was identified, active motor threshold (AMT) was obtained, identified as the minimum amount of stimulation required to trigger a muscle contraction in the activated abductor pollicis brevis. Participants were instructed to rest their right forearm on the arm of the chair while engaging their thumb in a “thumbs up” sign. Single pulses were delivered over the motor hotspot at varying intensities indicated by computerized Adaptive PEST for TMS tool (Dissanayaka et al., 2018; https://www.clinicalresearcher.org/software.html) while monitoring for a visual thumb twitch. Contraction was confirmed via the presence of a motor evoked potential, as measured by electrodes placed on the belly of the corresponding muscle. AMT was determined at each stimulation session, prior to delivery of active or sham TBS.

After session-specific AMT was determined, fiducial markers were placed and mapped to the individual’s head for precise navigation. MRI-guided Localite Neuronavigation software was used to align markers with individual anatomical T1-weighted images. This software allows for accurate identification of the left SMG, identified by a trained neuroanatomist as the apex of the ascending ramus of the superior parietal gyrus.

All participants received a minimum of four sessions (two treatment days) of sham stimulation consisting of active stimulation delivered to the SMG via a padded, 180-degree inverted Cool-B65 liquid cooled coil. After the initial baseline sham sessions, start day of active stimulation was pseudorandomized across participants within a given cohort. The first active stimulation session was administered during the first session of the assigned treatment day. Both stimulation protocols involved the administration of 20, two second trains of 50 Hz stimulation triplets delivered every 10 s for a total of 110 s (Huang et al., 2005). Stimulation was delivered at 80% of session-specific AMT. During stimulation, participants were instructed to silently read a list of progressively longer regular words designed to activate the reading network.

#### 3.2.2 SRA corrective reading

The SRA *Corrective Reading* (SRA/McGraw Hill, 1999) decoding program, using those components focused on addressing reading accuracy and fluency, was administered directly following each stimulation session (twice per day) by a trained reading specialist. Participants worked one-on-one with the reading specialist for 30 min per instruction session immediately following their stimulation session. The program used strategies taught through direct instruction that tracked the participant’s oral reading performance from a moment-to-moment basis, and immediately corrected errors, while gradually increasing reading material difficulty. Reading specialists followed explicit scripts for each lesson which specified what to say, do, and how to respond to each prompt.

During the pre-treatment/screening phase participants were assigned a program level based on performance on the SRA *Corrective Reading* Decoding Placement Test, with individuals placing into level B2 (*n* = 6) or C (*n* = 8) qualifying for the accelerated treatment phase. Decoding level B2 is appropriate for individuals who have difficulty decoding, are below typical reading rate, confuse words with similar spellings, and/or make word- guessing mistakes. Decoding level C is best suited for readers who have mastered basic decoding/reading skills but struggle with multisyllabic words and accuracy when reading quickly and need to increase their reading fluency. Lessons were similar in structure between the two levels, with a focus on accuracy and fluency. Each lesson consisted of oral word-attack practice, oral text reading, and individual reading checkouts. Reading specialists corrected every error immediately using the correction procedures for word attack or story reading tasks. The word attack portion consisted of instruction on common sound or sound combinations and focused on building-up vowel conversions (swapping out a sound combination) or by adding affixes and writing complex words as root words. Paired oral text reading included selections of fictional stories with factual information containing new words from sources such as newspapers and magazines. Comprehension skills were assessed using oral comprehension questions regarding content contained in the passages.

Following the completion of all required components of each lesson within the SRA *Corrective Reading* program there was a ‘reading checkout’ completed by the reading specialist. During this checkout, the participant orally read a text passage and the instructor tracked errors and number of words read during the time period given. Instructions stated that: “we will do a two-minute timed reading on the following story. Start with the first word of the story – do not read the title. I’ll tell you when to start and stop. Your goal is to read as many words as possible with as few errors as possible”. From each of these reading passages a measure of words read per minute and total number of errors made were recorded.

#### 3.2.3 Procedural fidelity

Stimulation procedures were kept consistent across stimulation sessions, participants, and cohorts. To account for normal variability in cortical excitability within individuals across time which may impact stimulation effectiveness, time of stimulation was kept consistent across sessions for a given participant. Session-specific AMT was also obtained at each session to ensure appropriate stimulation intensity, with motor hotspot confirmed prior to each session. Participants were instructed to reframe from significant changes in daily routine (i.e., caffeine/alcohol consumption, medication adherence, sleep patterns) that might impact cortical excitability and were screened for changes at the beginning of each treatment day. The use of individual anatomically defined stimulation targets guided by neuronavigation ensured consistency of stimulation site. Target site was saved prior to first stimulation session and kept consistent across proceeding sessions. Target site was also indicated on cap with mark serving as a locational auxiliary in the event of neuronavigation interruption. A member of the research team was also responsible for assuring participant adherence during stimulation including maintaining appropriate posture and reading of word list. Accuracy/fidelity ratings and comments were noted by two members of the research team (TBS technician and in-room assistant) after each participant session.

The use of the SRA *Corrective Reading* program was selected due to its explicit and well-validated directed instruction (Hempenstall, 2008; Flynn et al., 2012). Instruction is scripted to ensure that each session and reading specialist adheres to the same instructions and corrective language scripts throughout. Each lesson consisted of 3 parts: Word Attack, Story Reading and the Reading Checkout (note that because the instruction was conducted one-on-one, signals for choral responding, point system for motivation and individual accountability, peer checkouts, workbook exercises, and mastery tests typically used in group instruction settings were not used), and the basic steps are the same for all examples of a given part. As example of the level of explicit scripting, the Word Attack correction procedure included: “The word is _____. What word? (participant repeats). Spell _____. (participant spells while looking at the word). What word? (participant repeats), Go back to the first word in the row/column” (SRA/McGraw Hill, 1999). To ensure procedural fidelity, each reading specialist received 3 h of training from an experienced and previously SRA trained instructor who practiced with them until they were ‘fluent’ in the program scripting and expectations. Each reading specialist was a state-licensed educator or speech-language pathologist with years of experience and pedagogical training in literacy instruction. Reading specialists underwent training to ensure that they were able to perform online error correction as required by SRA in real time. Audio recordings of training and early sessions were reviewed to ensure consistency and fidelity of scripted instruction. Scripted instruction books were used consistently across participants with word-for-word scripted language for each session. In addition, all reading checkouts were recorded and reviewed to ensure instruction fidelity and to provide an auditory record of the participants’ performance for accurate data recording. Finally, at the end of each session, instructors kept a log for each participant’s session regarding the exact lessons completed, noting any exceptions made to the program scripting or student specific struggles/issues during the session. Logs and checkouts were reviewed by the instructors at the end of each day to note any deviations from protocol. All participants completed each 30-min session with no noted deviations from protocol. While participants may have completed a different number of raw lessons per day, they all received the same time-length of instruction. Number of lessons completed over the course of the program is noted in Table 2. To control for potential systematic individual instructor effects during the instructional program, including any minor fidelity differences, two different reading specialists were utilized in each cohort and were randomly assigned each day to a given participant’s morning or afternoon session so that no participant had the same teacher for both sessions within a day. In a few rare instances of reading specialist absence, all participants worked with the same specialist during both sessions of a given day.

**Table 2:** Summary of participant adherence and treatment session completion.

Case number	Cohort	Total baseline sessions	Total experimental sessions	Total makeup sessions	Total SRA lessons completed	Deviations
Case 1	1	4	16	2	57	N/A
Case 2	1	6	14	2	50	N/A
Case 3	1	20	0	0	53	Sham only due to stimulation intolerance
Case 4	2	8	12	0	82	N/A
Case 5	2	4	16	0	65	N/A
Case 6	3	6	14	2	59	N/A
Case7	3	4	16	2	55	N/A
Case 8	3	8	7	1	65	5 instruction-only sessions after stimulation intolerance
Case 9	5	6	14	2	46	N/A
Case 10	4	8	12	2	36	N/A
Case 11	4	6	14	0	42	N/A
Case 12	5	4	4	N/A	19	Discontinued due to excessive absences
Case 13	4	4	16	4	53	N/A
Case 14	5	20	0	4	53	Instruction only due to TBS contraindication

### 3.3 Data processing and visualization

The single-case reporting guidelines in behavioral interventions (SCRIBE) describes behavioral outcome variables as primary outcome variables and generalization variables, with generalization variables used to support the external validity of the study (Tate et al., 2016). Both primary and generalization variables are assessed at all phases of the protocol and reported, in line with best practices for evaluating behavior change (Schlosser and Braun, 1994).

#### 3.3.1 Standardized measures

All standardized assessments used during the screening/pre-treatment and post-treatment phases were reviewed and double scored by two independent, trained personnel. During data collection, each assessment was administered by a trained member of the research team, who scored in real-time, in line with standardized testing procedures. Following initial data collection, the individual who administered the assessment then reviewed audio files to confirm scoring. Following first scoring procedures, an additional trained personnel member who was not involved in the initial administration conducted an independent second scoring of all assessments. In the event of disagreement between first and second rater, a third rater would conduct an independent review and consult with the administrator for final scoring approval. For all WJ-IV subtests, raw scores were input into the WJ-IV online scoring system (https://riversideinsights.com/clinical-assessments/wj-v) which produced standardized subtest scores as well as composite scores including Broad and Basic.

#### 3.3.2 Daily oral reading fluency

ORF assessments were administered by trained study personnel who scored in real-time as passages were read aloud by the participant. Audio recordings were also completed during data collection sessions. Following assessment administration, the rater reviewed recordings to confirm initial scoring. Errors such as mispronunciations, substitutions, omissions, reversals, hesitations of longer than 3 s, and skipping of words were collected. Total word count was also noted and used in calculation of WCPM. A second trained personnel member who was naive to initial administration reviewed audio files and confirmed initial scoring. After first and second scoring was complete, an independent third person reviewed primary outcome scores for accuracy and consistency across raters. For this study, we consider ORF as our primary outcome variable as it assessed both fluency and accuracy, primary targets for reading instruction. Both on-level and above level ORF were included to addresses potential concerns regarding possible floor/ceiling effects.

#### 3.3.3 Expanded PD and OA tasks

PD and OA data were organized using in-house R scripts into targets (pseudohomophones in PD task and real words in OA task) and foils (nonwords in PD and OA tasks). Accuracy and median reaction time for correct responses were calculated. Responses <200 ms were not included in analysis to account for overshoots from previous trials. Median response time for correct responses in all conditions, target conditions and foil conditions were analyzed to calculate average, standard deviation, slope, and trend-stability envelope for the pre-treatment, baseline phase, experimental phase and post-treatment phases (Manolov et al., 2014). PD and OA were considered generalization variables as prescribed by the SCRIBE guidelines (Tate et al., 2016).

#### 3.3.4 fMRI visualization

fMRI processing was performed using FEAT (FMRI Expert Analysis Tool), part of the Functional Magnetic Resonance Imaging of the Brain FMRIB’s Software Library (FSL, https://fsl.fmrib.ox.ac.uk/fsl/docs/#/), version 6.0. Pre-statistical processing included: brain extraction using BET (Smith, 2002), motion correction by FMRIB’s Linear Image Registration Tool; MCLFIRT (Jenkinson et al., 2002), spatial smoothing using a Gaussian kernel of FWHM 5 mm and high-pass temporal filtering. Each participant’s fMRI was co-registered (12 degrees of freedom) to their T1-MPRAGE image, and this latter (T1-MPRAGE) was co-registered to the Montreal Neurological Institute standard space (MNI 152_2mm_brain template) using FLIRT (FMRIB’s Linear Image Registration Tool) (Jenkinson and Smith, 2001; Jenkinson et al., 2002), which was then further refined using FNIRT nonlinear registration tool in FSL. Registration quality was checked by visual inspection.

A whole-brain analysis based on the general linear model (GLM) was performed after preprocessing, with local autocorrelation correction (Woolrich et al., 2001). The first-level GLM included regressors for each condition and task run (3 runs for Fast-Loc and 2 runs for PID). Six motion parameters plus extended parameters – using MCFLIRT – were included in the model as regressors of no interest. Conditions for each task were modeled using a double-gamma hemodynamic response. Additionally, temporal filtering was applied, and temporal derivatives of each condition were also included for the model to best fit the time course of the actual data acquisition. A second-level GLM used a fixed-effect model to average parameter estimations across runs within each subject. To average over similar responses, the PID analysis was restricted to functional activation of spoken and written words for the mismatching trials (25 trials in total for each mismatching condition). The Fast-Loc task was restricted to the standard trials of the following stimulus type: false font, pseudowords, unrelated words, words with shared orthography and shared phonology, words with shared orthography but different phonology and semantically related words. Z (Gaussianised T) statistic images for each individual case were thresholded at *p* < 0.01, uncorrected. Main significant clusters were inspected in FSLeyes atlas view based on the MNI coordinates of the peak activation for each cluster. The Harvard-Oxford atlases – cortical and subcortical – were used for cluster location and inspection. Cerebellar atlas in MNI152 space after normalization with FLIRT was used for cerebellar activations.

#### 3.3.5 Visualization of ORF, PD, and OA tasks

##### 3.3.5.1 Normalized mean phase difference visualization

ORF, PD, and OA task outcomes were assigned to baseline or experimental phase based on time point of data collection. Odd and even sessions represented morning and afternoon data collection sessions, respectively. Mean and slope were calculated for each phase for each participant for traditional visual inspection of the data.

##### 3.3.5.2 Slope and level change visualization

Because participants received twice daily SRA instruction in the baseline phase, baseline reading performance was not expected to be stable. To estimate the experimental-phase deviations from the projected baseline, we calculated slope and level change as an additional analysis to supplement traditional visual analysis (Solanas et al., 2010). By detrending the data, we can partially isolate the effect of aTBS from SRA instruction.

SLC is a modified form of traditional regression techniques often applied to single-subject data such as split-middle trend envelopes. Analysis of SLC found it performed well on bias and error while split-middle performed sub-optimally (Manolov, 2023). In split middle trend envelopes, baseline trend is projected into the experimental phase. If data points fall outside of the envelope, it is considered outside of the predicted trend of the baseline phase (Manolov et al., 2014). In SLC, session by session trend estimates are computed and subtracted from subject and session specific experimental phase data. When analyzing SLC results, it can be considered conceptually similar to trend envelopes. A positive slope or level change value conceptually equates to data points outside of the top bound of the envelope. A negative slope or level change value would equate to data points within or below the bounds of the envelope. Detrended data should therefore not be interpreted as raw directional change but as change relative to baseline. A negative value does not indicate performance decreased in raw terms, but suggests that the treatment phase trend was flatter or lower than the expected trajectory based on the baseline trend.

Detrending was performed using *R* scripts modified from Supplementary Appendix B of Solanas et al. (2010). First, average difference was calculated between data points in the baseline phase. To detrend the baseline phase, the baseline slope multiplied by the session number was subtracted from each observed value. For the experimental phase, the same calculations were performed with session number beginning at 1 at the phase boundary so that time remained continuous across the phase boundary. With beta (*β*) removed from the experimental phase, any remaining change in level or slope can be attributed to the experimental condition. Reaction time data signs were reversed for readability, to represent a decrease in reaction time as a positive change.

## 4 Preliminary findings

### 4.1 Program completion

Thirteen of 14 participants successfully completed all 20 sessions of study participation. Eleven participants were assigned to baseline/experimental randomization (Table 2). One participant received baseline only (sham + instruction) across all sessions due to anxiety-related intolerance to active stimulation. One participant received instruction only (no stimulation) due to a benign neuroanatomical TBS contraindication identified following pre-treatment MRI. One additional participant received a combined 15 baseline and experimental sessions and then completed five instruction-only sessions after reporting discomfort associated with active stimulation. One participant was disqualified for excessive absences following a combined 8 baseline and experimental sessions. Eight participants missed 2 or less treatment days (max 4 sessions) due to illness or scheduling conflicts for which experimental makeup sessions were completed at the end of the accelerated treatment period, prior to the post-treatment stage. One of these participants completed a morning treatment session but was unable to complete the afternoon session. The missed afternoon session was completed at the end of the treatment period, consistent with makeup procedures, at the time consistent with previous afternoon sessions.

### 4.2 Adverse events

Possible adverse events, including but not limited to headache, muscle ache, fatigue, and seizure were assessed at the end of each treatment day as well as following pre- and post-treatment days. A structured adverse event interview questionnaire was completed by a member of the research team and reviewed by the study nurse. The study nurse evaluated participant safety and comfort after consultation with participant to establish need for discontinuation and/or withdrawal. Overall, combined treatment was well tolerated with only two participants being withdrawn from the aTBS portion of treatment; one associated with increased anxiety due to muscle contraction/jaw twitch during active stimulation, the other due to persistent headache following active stimulation. No seizures were observed or reported in association with study involvement. The most common side effect was headache and/or fatigue which was reported following both baseline (4 participants) and experimental (7 participants) treatment days. Transient muscle or jaw twitch was also reported during the experimental treatment phase (4 participants). Two participants reported transient ringing in their ears following stimulation. Two participants reported dizziness or light-headedness following either the pre- or post-treatment MRI scan.

Adverse events and negative impact on behavioral outcomes were also monitored by an external data and safety monitoring committee. The three-person independent panel reviewed weekly adverse event and behavioral data reports. No unanticipated problems or serious adverse events were reported. No stopping rules were triggered during the course of the study.

### 4.3 Daily oral reading fluency

#### 4.3.1 ORF on-level

**Figure 3:**
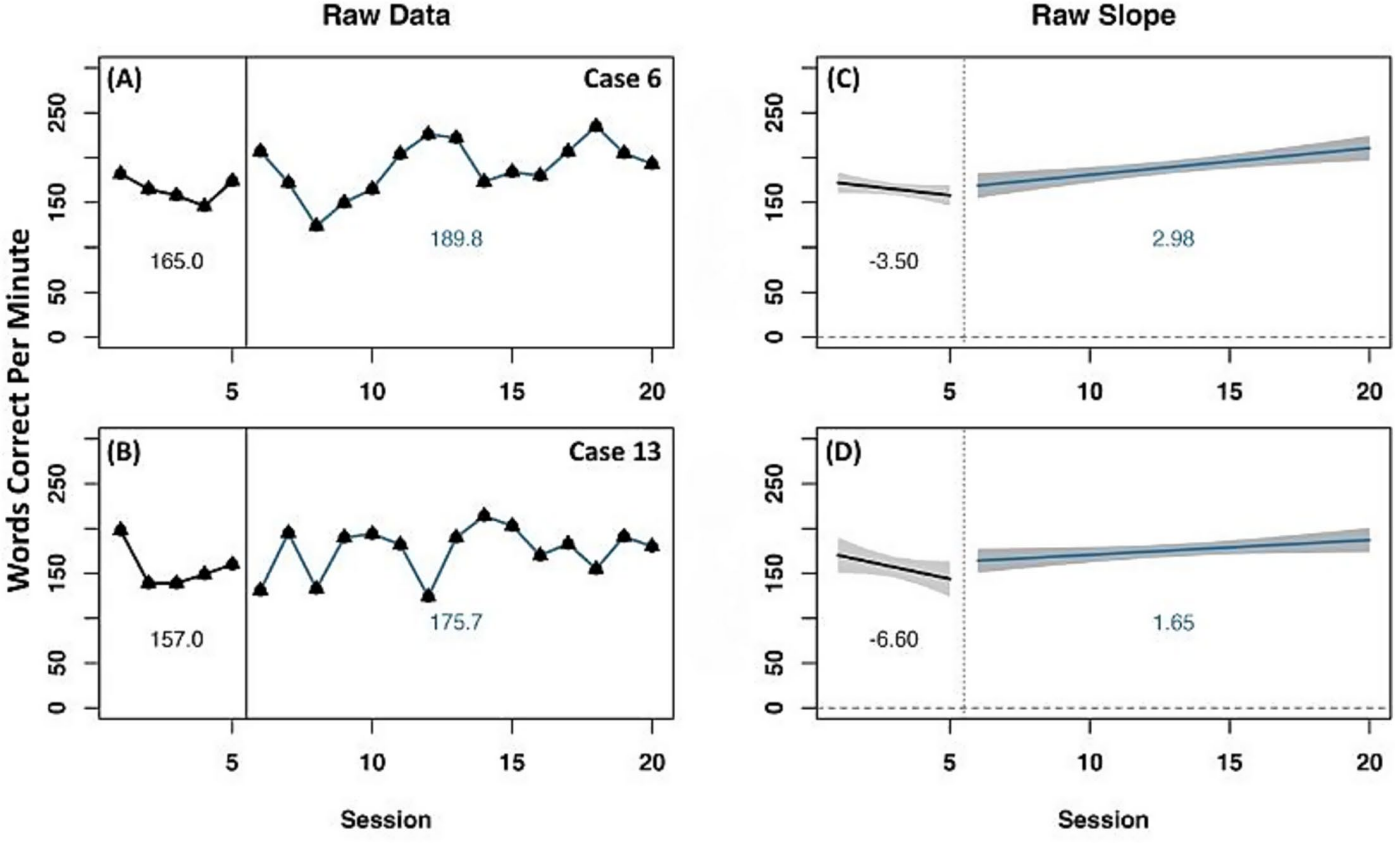
Representative case series of raw data words correct per minute for on-level oral reading fluency measure for strong responders. **(A,B)** present mean differences between baseline and experimental phases for corresponding cases. **(C,D)** present slope differences between baseline and experimental phases for corresponding cases. Vertical session split represents point of transition between phases. Black line prior to session split represents baseline phase. Blue line after session split represents experimental phase.

A representative selection of cases is presented to describe experimental treatment outcomes for the on-level ORF measure. Two cases (6 and 13) were identified as having reliable increase in WCPM across raw data mean and slope change, as well as detrended data (Figure 3). Both cases had a negative slope in the baseline phase and positive slope in the experimental phase. When data were detrended, both cases had a positive slope and level change (Table 3), indicating a reliable effect of experimental phase treatment.

**Table 3:** Slope and level change of detrended data for representative cases.

Case number	On-level ORF WCPM		Above-level ORF WCPM		Phonological decoding task		Orthographic awareness task	
	Slope change	Level change	Slope change	Level change	Slope change	Level change	Slope change	Level change
Case 2	−0.8	−16.7	6.5	16.4	33.8	392	36.2	92
Case 5	5	15.1	6.0	6.9	−54.5	−227.8	−97.3	−17
Case 6	1	37.8	−3.6	2.8	26.8	81.9	−18.5	−33.7
Case 7	1.4	−26.5	−10.9	−34	63.5	199.1	−52	−45.9
Case 10	−1.6	−2	−4.4	−1.5	NA	NA	20.4	−30.8
Case 11	3	−8.7	−1.7	22.9	−33.2	−121.9	NA	NA
Case 13	13	22.6	7.5	9.05	−54.9	195.6	14.4	−68.5

ORF, oral reading fluency; WCPM, words correct per minute. Values represent change at the baseline phase/experimental phase transition. For WCPM, positive values represent more WCPM or faster WCPM change at the phase transition. For reaction time (OA and PD), signs have been reversed so that improvement in reaction time is represented as a positive value.

Five representative cases were identified as having change on at least one of the three metrics for on-level ORF (Supplementary Figure 1). Two cases (2 and 5) showed minimal change in raw mean WCPM from baseline to experimental phase. Case 2 had a positive slope during both phases but showed a flatter slope during the experimental phase. Case 5 had a large negative slope during the baseline phase with a flatter, but still negative slope, during the experimental phase. Detrended data for case 2 indicated a negative slope and level change suggesting no effect of experimental phase. Case 5 had a positive change in both slope and level, indicating a significant effect of experimental phase treatment. Case 7 had a raw mean WCPM increase from baseline to experimental phase. In this case, there was a positive slope in both phases with a minimally steeper slope during the experimental phase, however detrended data indicated a positive slope change and negative level change. Two cases (10 and 11) had change in raw mean WCPM and no significant change in level or slope for detrended data. In both cases, there was a steeper positive slope for the baseline phase compared to the experimental phase with raw data showing increased variability between morning and afternoon assessment (see Supplementary Figure 1) during the experimental phase.

**Figure 4:**
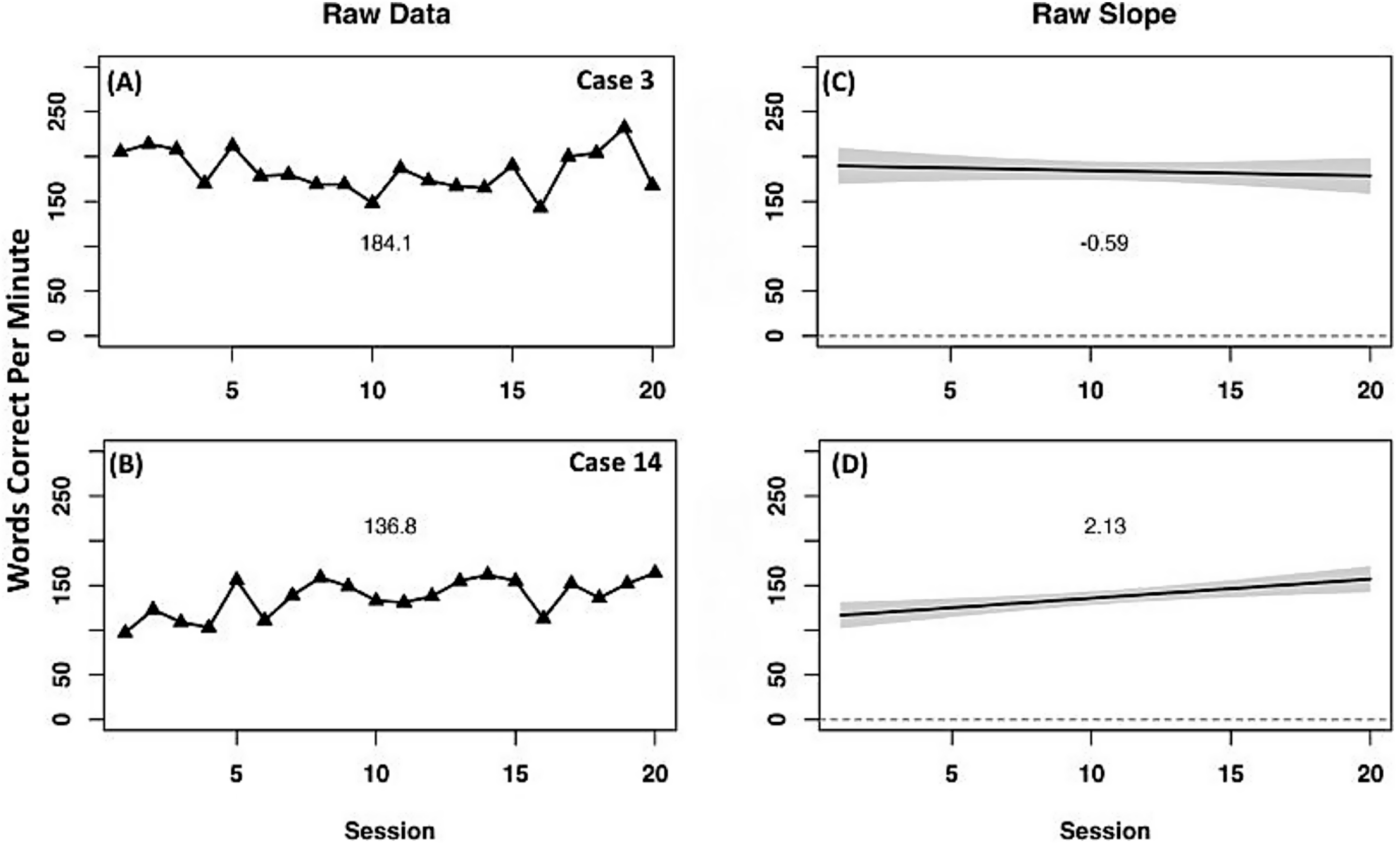
Case series of raw data words correct per minute for on-level oral reading fluency measure for comparison cases. **(A,B)** present mean performance across treatment for corresponding cases. **(C,D)** present slope of performance across treatment for corresponding cases.

The two comparison cases had differing results (Figure 4). Case 3 (instruction + sham) had a small negative slope with minimal change across time, indicating a negligible decrease in WCPM. Case 14 (instruction only) had a positive slope and improved in WCPM across the instruction period.

#### 4.3.2 ORF above-level

**Figure 5:**
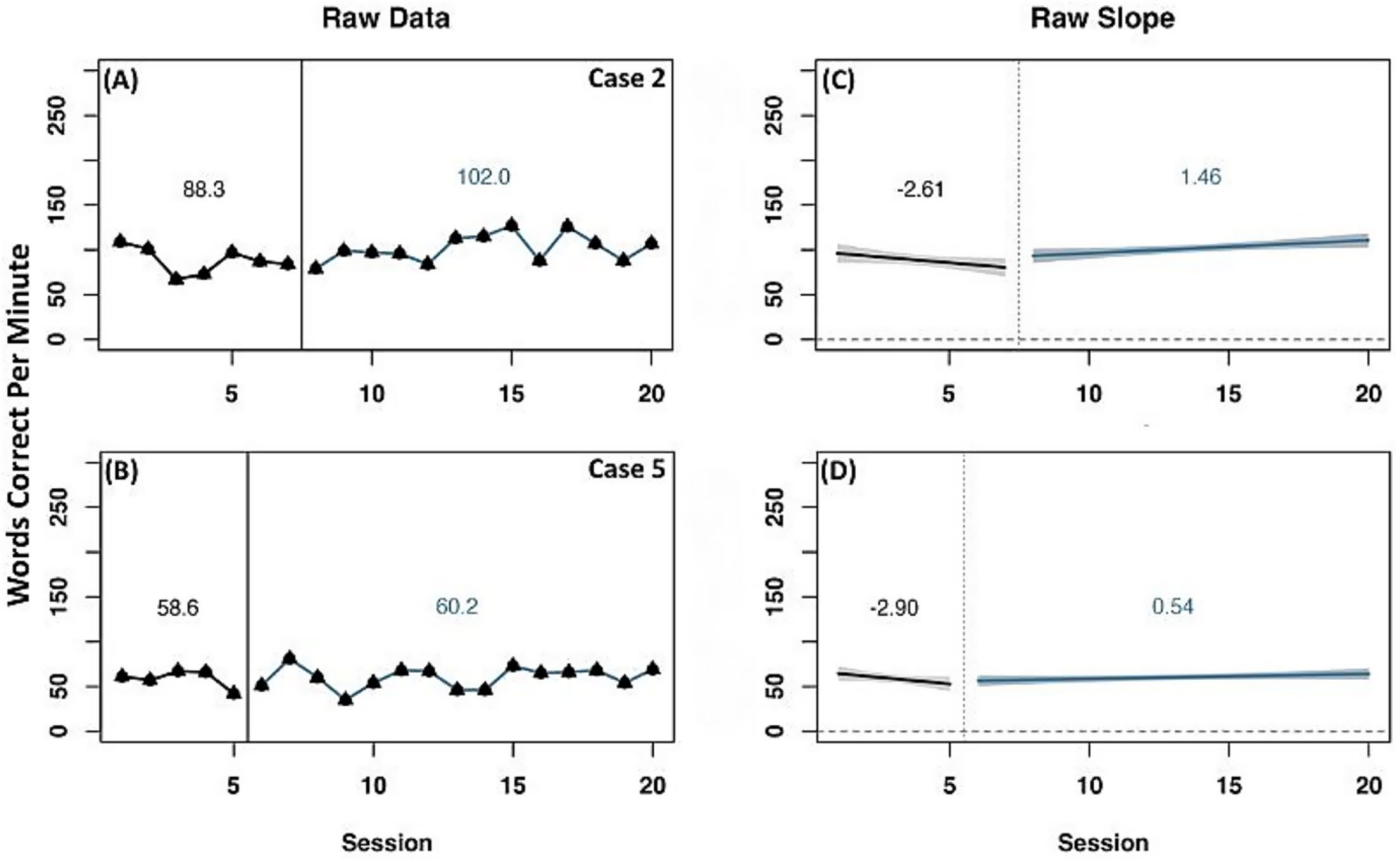
Representative case series of raw data words correct per minute for above-level oral reading fluency measure for strong responders. **(A,B)** present mean differences between baseline and experimental phases for corresponding cases. **(C,D)** present slope differences between baseline and experimental phases for corresponding cases. Vertical session split represents point of transition between phases. Black line prior to session split represents baseline phase. Blue line after session split represents experimental phase.

Similar to ORF on-level, two cases (2 and 5) were identified with a reliable increase in WCPM for the ORF above-level measure across raw data mean and slope change, as well as detrended data (Figure 5). Both cases had a negative slope during the baseline phase and a positive slope during the experimental phase. When data were detrended, both cases had a positive slope and level change, indicating a reliable effect of experimental phase treatment.

Five representative cases showed change on at least one data measurement method for the above-level ORF measure (Supplementary Figure 2). Case 6 showed change in raw mean WCPM from the baseline to experimental phase, with a negative slope during baseline and a positive slope during the experimental phase however, detrended data indicated a negative slope change with a positive level change. Case 7 had an increase in raw mean WCPM from the baseline to experimental phase. In this case, there was a positive slope in both phases with a more flattened slope during the experimental phase, however detrended data indicated a negative slope and level change, indicating no effect of experimental phase. Two additional cases (10 and 11) had change in raw mean WCPM and a steeper positive slope during baseline compared to the experimental phase. For case 10, detrended data showed a negative change in slope and level while case 11 detrended data represented a negative change in slope but a positive level change. Conversely, no significant change in raw mean WCPM was observed for case 13 with a negative slope at baseline and a positive slope during the experimental phase. Detrended data for this case indicated a positive change in slope and level, suggesting an effect of experimental phase.

**Figure 6:**
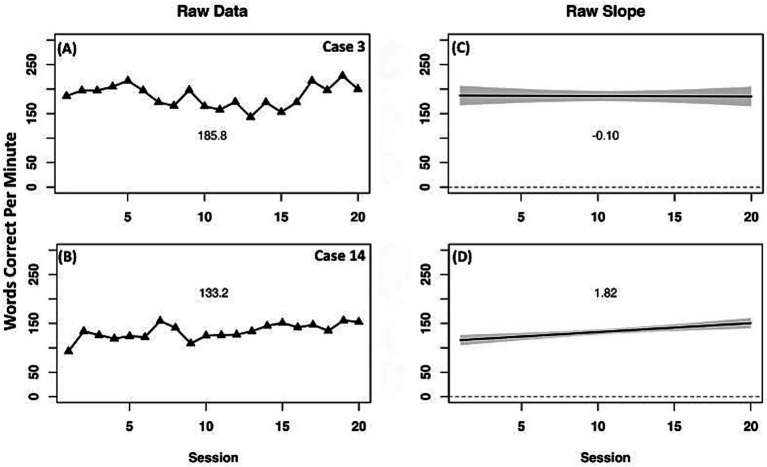
Case series of raw data words correct per minute for above-level oral reading fluency measure for comparison cases. **(A,B)** present mean performance across treatment for corresponding cases. **(C,D)** present slope of performance across treatment for corresponding cases.

Consistent with their results on the ORF on-level measure, the two comparison cases had opposing outcomes (Figure 6). Case 3 had a small negative slope with minimal change across time, indicating a negligible decrease in WCPM. Case 14 had a minimal improvement in raw WCPM and a positive slope indicating a small improvement in above-level ORF across the instruction period.

### 4.4 Expanded phonological decoding task

**Figure 7:**
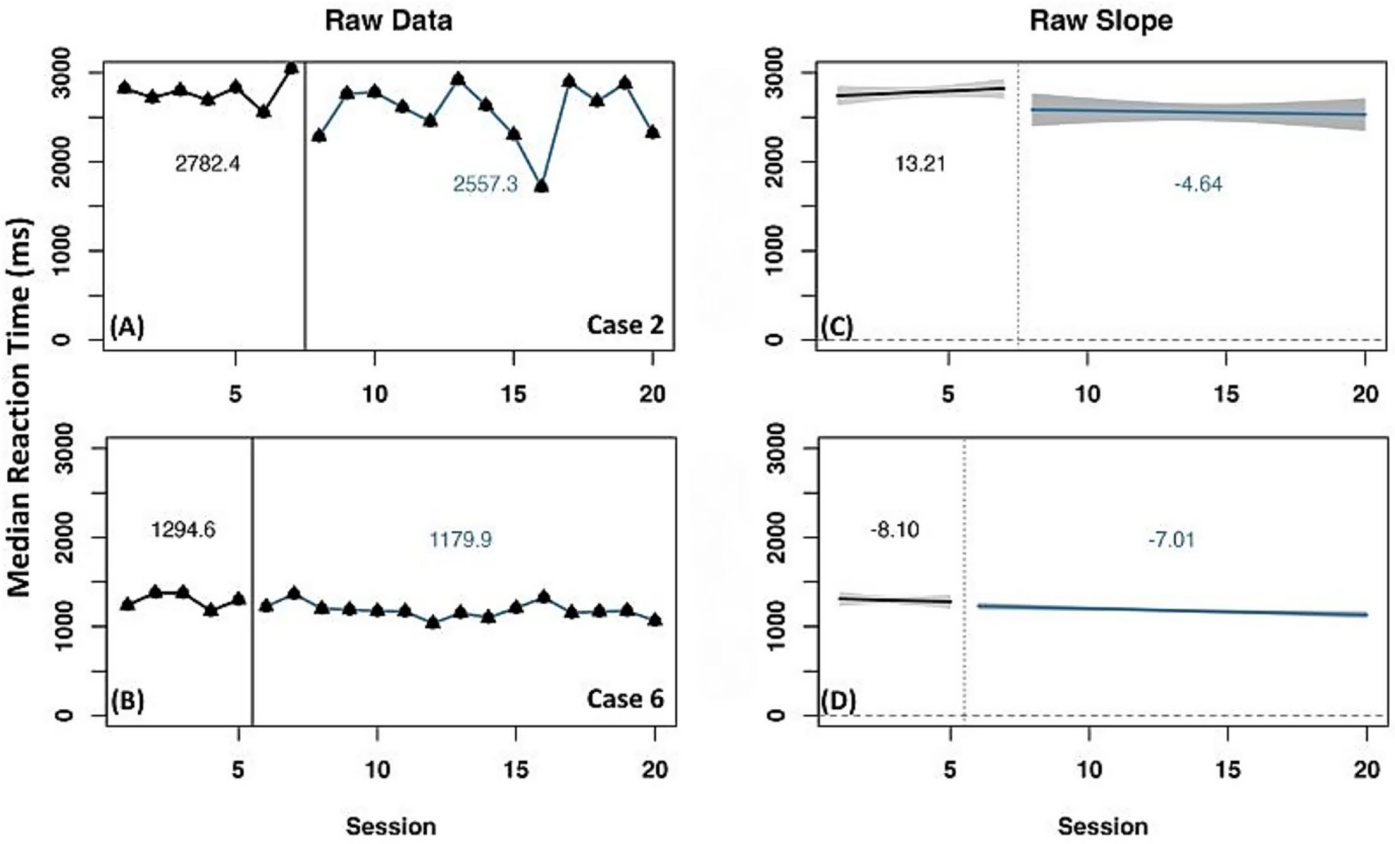
Representative case series of raw data median reaction time in milliseconds for expanded phonological decoding measure for strong responders. **(A,B)** present mean differences between baseline and experimental phases for corresponding cases. **(C,D)** present slope differences between baseline and experimental phases for corresponding cases. Vertical session split represents point of transition between phases. Black line prior to session split represents baseline phase. Blue line after session split represents experimental phase.

Results for the PD task are presented for the same representative cases as above. Two cases (2 and 6) had reliable improvement in phonological processing skills, represented by a decreased median reaction time, as indicated by consistent results across all three measurement methods (Figure 7). Both cases had a raw mean reduction in median reaction time from the baseline to experimental phase. Case 2 had a positive (slower) slope in the baseline phase with a negative (faster) slope in the experimental phase. Case 6 had a faster slope in both the baseline and experimental conditions. Detrended data for both cases also indicated a positive slope and level change, suggesting an effect of experimental phase treatment.

Faster raw median reaction time was seen in most other cases for the baseline phase, with evidence for improvement in both baseline and experimental phases (Supplementary Figure 3). One case (10) did not produce reliable data due to technical malfunction during data collection. For case 5, mean raw median reaction time was faster in the experimental phase however there was a less steep negative slope in the experimental phase as well as a negative slope and level change in the detrended data. Raw data for case 7 indicated slower median reaction time as well as a flatter slope in the experimental phase compared to the baseline phase. After detrending, there was a positive slope and level change for this case, suggesting an effect of experimental phase treatment. Case 11 showed the opposite outcome with mean raw data indicating faster median reaction time as well as a steeper slope in the experimental phase but a negative slope and level change in the detrended data. Case 13 also had faster mean raw data but a flatter slope in the experimental phase however detrended data indicated a negative slope and positive level change, suggesting little to no effect attributed to experimental phase treatment.

**Figure 8:**
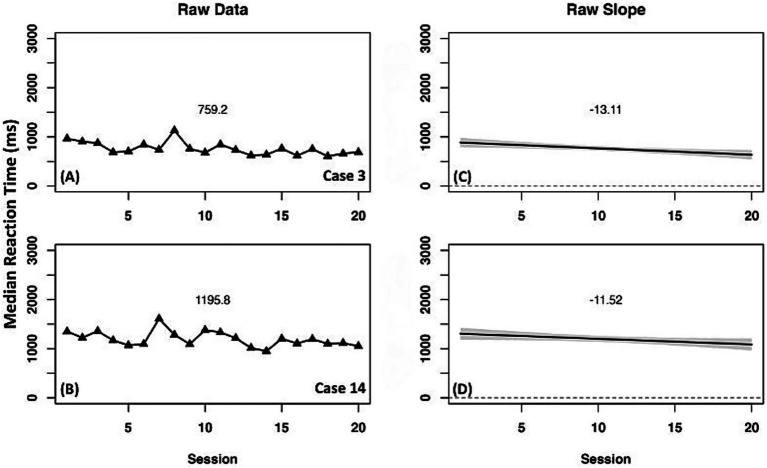
Case series of raw data median reaction time in milliseconds for expanded orthographic awareness measure for comparison cases. **(A,B)** present mean differences between baseline and experimental phases for corresponding cases. **(C,D)** present slope differences between baseline and experimental phases for corresponding cases.

Comparison cases (3 and 14) had minimal raw speeding of median reaction time with a negative slope indicating small, steady improvement in phonological processing across treatment (Figure 8).

### 4.5 Expanded orthographic awareness task

**Figure 9:**
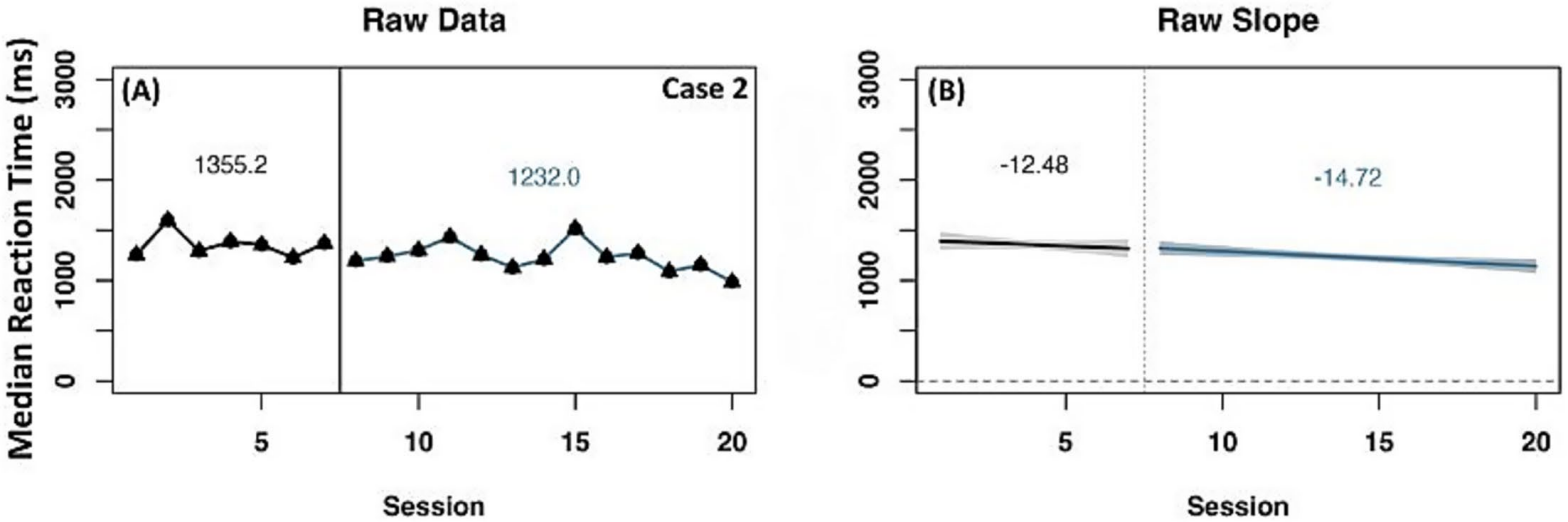
Representative case series of raw data median reaction time in milliseconds for expanded orthographic awareness measure for the strong responder (case 2). **(A)** presents mean differences between baseline and experimental phases. **(B)** presents slope differences between baseline and experimental phases. Vertical session split represents point of transition between phases. Black line prior to session split represents baseline phase. Blue line after session split represents experimental phase.

Only one case (2) showed reliable change across all measurement methods on the OA task (Figure 9). In this case, mean raw median reaction time was faster and there was a sleeper negative slope in the experimental phase compared to baseline. A positive slope and level change was also observed in the detrended data indicating improvement specific to the experimental phase treatment.

Other representative cases were identified with raw data suggesting faster reaction time across phases but a negative change in slope and level in detrended data, indicating no specific improvement contributed to experimental phase (Supplementary Figure 4). One case (11) did not produce reliable data due to technical malfunction during data collection. Two cases (5 and 6) had speeding of raw median reaction time with a negative slope in the baseline and a less negative slope in the experimental phase, indicating a general overall improvement in reaction time. However, there was a negative slope and level change when detrended, indicating no specific improvement attributed to the experimental phase. From baseline, Case 6 was within the expected reaction time range for that of unimpaired individuals therefore lack of change in this case may be more representative of a ceiling effect of task performance. Case 7 had slower mean raw median reaction time and flatter slope in the experimental phase, as well as a negative slope and level change after detrending. Two cases (10 and 13) had a speeded mean raw performance on the OA task with a negative slope in the baseline and a less negative slope in the experimental phase. Detrended data for these cases indicated a positive slope and negative level change. In both cases, a negative level change may be indicative of a ceiling effect of task performance reached during the experimental phase as mean raw median reaction time trends within the range of typical performance.

**Figure 10:**
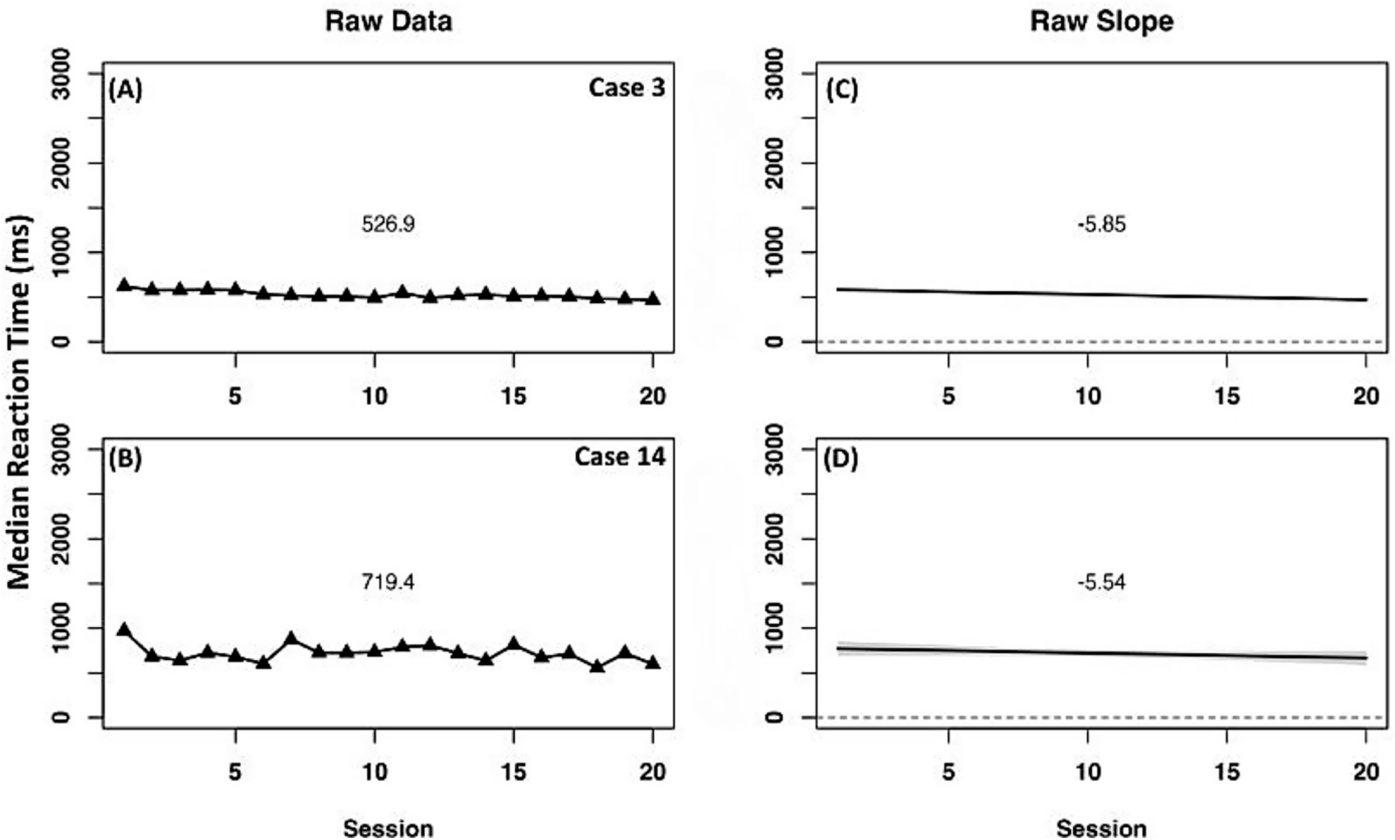
Case series of raw data median reaction time in milliseconds for expanded orthographic awareness measure for comparison cases. **(A,B)** present mean differences between baseline and experimental phases for corresponding cases. **(C,D)** present slope differences between baseline and experimental phases for corresponding cases.

Case 3 and Case 14 had negative slopes indicating small and incremental improvement in reaction time (Figure 10). This provides evidence for a small effect of instruction on OA task performance.

### 4.6 Task-based fMRI

#### 4.6.1 Fast-Loc task

**Figure 11:**
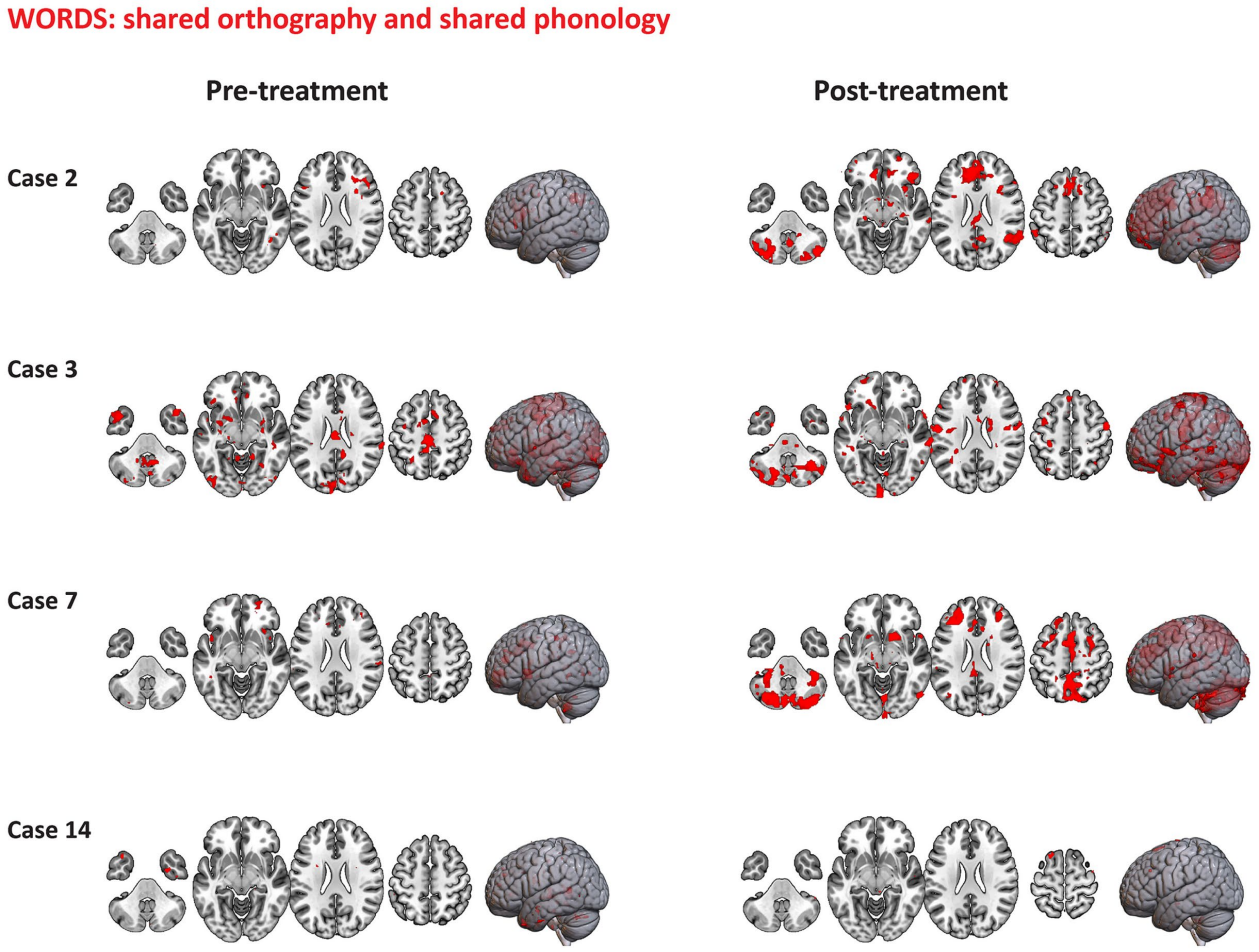
Representative case series of functional activation pre- and post-treatment for words with shared orthography and shared phonology condition during the Fast-Loc task. Activation regions (*p* < 0.01, uncorrected *Z* values) are presented in neurological convention.

Visualization of representative case series of functional activation pre-and-post treatment for words (with shared orthography and shared phonology) are presented in Figure 11. Overall, cases 2 and 7 are representative of those who underwent combined aTBS and reading instruction displaying more distributed areas of activation post-treatment (*p* < 0.01, uncorrected) during the processing of words (with shared orthography and shared phonology) in comparison to pre-treatment. The primary identified clusters showing activation post-treatment included voxels across bilateral frontal regions, cerebellum, thalamus, superior parietal lobule and lateral occipital cortex in these cases. Other identified clusters that showed increased activation following treatment were observed in the bilateral SMG, angular gyrus and inferior frontal gyri (pars triangularis and pars opercularis). Case 3, who received only baseline treatment (sham stimulation + instruction), seems to engage relatively similar regions pre- to post-treatment, while Case 14 who received instruction only, showed minimal activation in response to written words both pre-and post-treatment.

**Figure 12:**
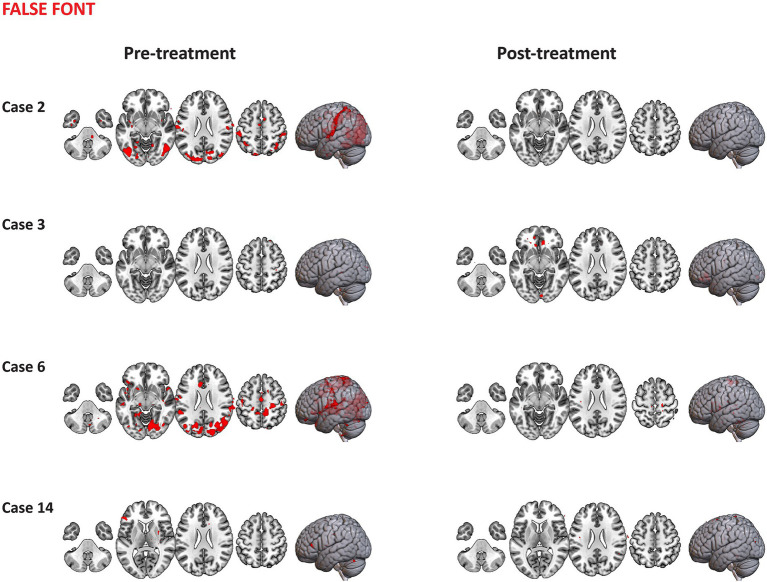
Representative case series of functional activation pre- and post-treatment for false font condition during the Fast-Loc task. Activation regions (*p* < 0.01, uncorrected *Z* values) are presented in neurological convention.

Different activation patterns during the processing of false font are displayed in Figure 12. Those participants who received combined TBS and reading instruction (e.g., Cases 2 and 6) showed distributed areas of activation pre-treatment for false fonts. The primary clusters identified were distributed across temporooccipital regions including the inferior and middle temporal gyri, occipital fusiform gyrus and lateral occipital cortex with other clusters included SMG and postcentral gyrus. Minimal activation in these regions was observed post-treatment. Comparison cases 3 and 14 showed minimal areas of activation pre- or post-treatment.

#### 4.6.2 PID task

**Figure 13:**
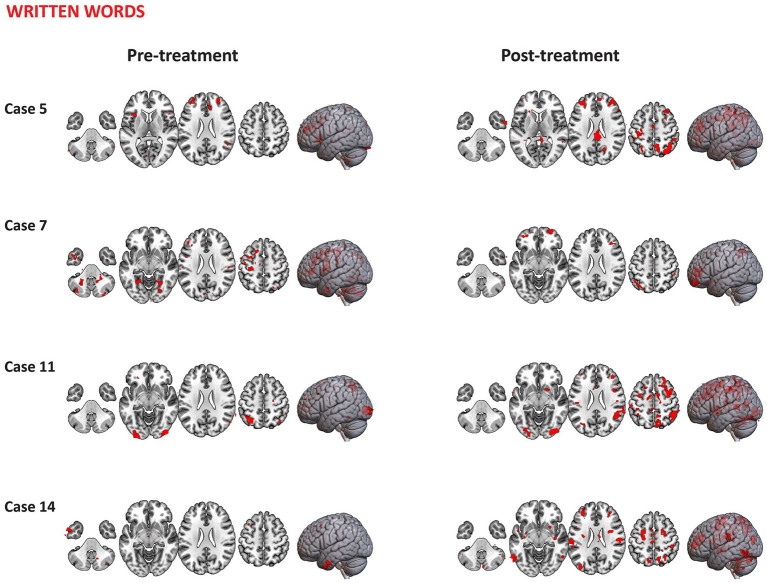
Representative case series of functional activation pre- and post-treatment for written words during the PID task. Activation regions (*p* < 0.01, uncorrected *Z* values) are presented in neurological convention.

Visualization of representative cases of functional activation pre-and-post treatment for written words are displayed in Figure 13. Participants who received combined TBS and reading instruction (e.g., cases 5, 7 and 11) showed distributed areas of activation pre-and post-treatment. These cases displayed slightly more regions of activation post-treatment in comparison to pre-treatment across frontal regions (including the superior frontal gyrus, the cingulate and paracingulate gyri) and the temporooccipital fusiform cortex. Case 14 who received instruction only, showed an increased number of regions with activation following treatment, with maximum peaks of activation within the main clusters distributed across the bilateral superior parietal lobule, lateral occipital cortex, precentral and postcentral gyri, superior frontal gyrus, SMG, inferior temporal gyrus (temporooccipital part) and cerebellum.

## 5 Discussion

Reading is a critical skill for one’s quality of life and health. Although DD is typically thought of as a disorder impacting educational outcomes in childhood, reading deficits associated with the most severe impairments often persist into adulthood. Despite this, few behavioral interventions have been successful in remediating this adult population (Greenberg et al., 2011). Inducing neuroplasticity with noninvasive neuromodulation prior to reading instruction may facilitate learning for those adults with DD. The methodology and preliminary findings reported here support the tolerability and feasibility of an accelerated treatment protocol of combined aTBS and reading instruction for young adults with DD. The aim of the current manuscript was to describe the methodology for this protocol and provide preliminary findings to increase adaptability and replicability. Previous research has indicated that a single session of transcranial magnetic stimulation can transiently modulate reading outcomes in both typically developing and reading impaired populations (Arrington et al., 2023). Our study provides proof-of-concept for a protocol combining multiple, accelerated sessions of TBS and reading instruction for the remediation of persistent DD.

We provide preliminary evidence supporting the feasibility and potential effectiveness of the current protocol to improve phonological processing and reading fluency in a sample of young adults with a history of developmental reading impairments. For behavioral outcomes, we present both raw and detrended data in an effort to identify a method for best representing trends in treatment outcomes across single subjects. We observed a general improvement in raw mean performance across tasks in most cases. In those participants where there was a positive slope and level change observed, evidence suggests an independent effect of combining instruction and stimulation, above instruction alone. This was most often the case for the ORF task, both on-level and above-level passages. This task is most closely related to skills specifically targeted by the reading program. Considering that one comparison participant (case 14) also had a positive slope across the treatment period, there is some evidence for an effect of instruction only. However, the other comparison participant (case 3) showed no significant improvement in ORF with a slight negative slope across treatment. Although both participants had a history of DD, case 3 was identified at screening as a less impaired reader than case 14 (SRA level C and B2, respectively). This may suggest that for those most impaired readers, particularly those with minimal previous behavioral intervention, accelerated instruction alone may have a positive impact on reading fluency.

Interestingly, the B2 representative cases which were presented here (2 and 5) had only marginal change in raw mean WCPM for both on-level and above-level passages but both cases had a positive slope and level change for above-level passages and case 5 had a positive change for on-level as well. This change would suggest a significant effect of combined TBS and reading instruction above instruction alone. The change from a negative slope during the baseline phase to a positive slope during the experimental phase, despite only incremental change in mean raw performance, may suggest that adding neuromodulation as an adjunct to reading instruction may be able to facilitate positive reading outcomes and that with additional treatment sessions meaningful behavioral change may occur. Previous evidence recommends completion of a minimum of 65 lessons from the SRA *Corrective Reading: Decoding* program in order to achieve improvement in targeted reading skills (Hempenstall, 2008), with largest effects noted after 70 contact hours of direct instruction (Lovett et al., 2000). In the current study, participants only received 20 contact hours with most coming close to but not meeting the recommended 65 program lessons (mean = 52.5 lessons). Alternatively, case 5 completed the total recommended number of lessons with only minimal change in raw reading performance. If the observed change in detrended data was due to the impact of lessons received rather than experimental effects then we would not except to see similar change in case 2 who completed only 50 SRA lessons. Additionally, one level B2 participant not presented in current representative cases (case 5) completed at total of 82 lessons. This participant had only minimal change in reading fluency measures but showed a change in slope for the reaction time measures (OA and PD) during the experimental phase. This participant was also randomized to the least amount of active stimulation sessions (*n* = 12). This may suggest that more sessions of active stimulation may have produced larger improvements in reading fluency. As such, the observed improvements in reading outcomes, even incremental, may be more indicative of the effects of combined treatment versus instruction alone. If minimal behavioral change was due to underdosing of instruction then we would expect to see larger effects of treatment in those with more lessons, however this is not consistently the case.

Additionally, preliminary visualization of functional neuroimaging data suggest an impact of combined treatment on the brain’s reading related networks that are distinct from reading instruction alone. Specifically, we observed increased activation for the processing of real words in both the Fast-Loc and PID tasks following combined treatment in regions within the reading network and other distant but functionally related cognitive and executive control regions. Consistent with these findings, several studies and meta-analytic work have reported significant activation of the cerebellum and thalamus during reading and language-related tasks (Stoodley and Stein, 2013; Martin et al., 2015; Alvarez and Fiez, 2018). Other upregulated brain regions observed in our study (e.g., cingulate and superior frontal gyrus), are known to be engaged in higher cognitive and executive control functions that would presumably support reading ability (Shaywitz et al., 2002; Buchweitz et al., 2009; Turker et al., 2023). Our preliminary findings also support previous work showing that repetitive neuromodulation paradigms are effective in modulating regions within functionally specialized networks but may also impact distal brain regions through network interactions (Sale et al., 2015; Herrera-Diaz et al., 2025).

For purposes of this manuscript, we present uncorrected results of representative cases from the shared orthography/shared phonology condition for visualization purposes, as this contrast seemed to be most reliable across participants and sensitive to treatment outcomes. An additional benefit of this approach is that it allows for presentation of primary outcome variables and generalization variables without the risk of multiple comparisons. For two participants who showed minimal behavioral response to combined treatment, we observed increased activation post-treatment for other types of words (case 1, unrelated words and words with shared orthography but different phonology; case 9 for semantically related words). This may suggest the detection of early network changes associated with the effects of combining TBS and reading instruction that have not yet manifested into behavioral change. This is consistent with past evidence suggesting that changes in functional activation are often observed prior to measurable behavioral change (Hartwigsen and Volz, 2021; Hartwigsen and Silvanto, 2023).

One of the comparison cases (14, instruction only) showed increased activation for written words during the PID task, but not during the Fast-Loc task. This is consistent with behavioral outcomes observed in the same participant such that improvement in word reading accuracy and fluency was observed in the ORF task but not in reaction time on the OA or PD tasks. This may be due, in part, to the sensitivity and complexities of task demands. The PID task is a slower task, allowing for more processing time which may be more consistent with the cognitive demands of the ORF task. These tasks that focus on accuracy and fluency may be more in line with the skills targeted by the SRA *Corrective Reading* program and thus may be more sensitive to change directly associated with instruction alone. Conversely, the Fast-Loc task as well as the PD and OA tasks require more automized word recognition and speeded processing. Both comparison cases (3 and 14) showed no observable change in brain activation on the Fast-Loc task post-treatment. While both demonstrated improvement in PD and OA task performance, this was minimal compared to others who received combined treatment. This may suggest that change observed in OA and PD task performance may be associated with learning effects specific to the tasks rather than meaningful behavioral change. This may also support previous evidence that demonstrates the impact of combined neuromodulation and task specific training on processing speed and neural efficiency (Luber and Lisanby, 2014; Curtin et al., 2019). Deficits in processing speed are often observed in DD (Fawcett and Nicolson, 1994; Stoodley and Stein, 2006), even in the absence of deficits in higher level component reading skills such as reading comprehension (de Oliveira et al., 2014). Our preliminary results may indicate that the use of neuromodulation to facilitate processing speed may be key in enabling networks supporting reading in those with persistent DD.

### 5.1 Limitations and future directions

The primary aim of the current manuscript was to present a structured methodological approach to highlight the feasibility and tolerability of a combined treatment paradigm for adults with DD. Presentation of preliminary findings are help illustrate potential outcomes and should be interpreted with caution as they may not generalize beyond what is presented here. It is also important to highlight that single-subject experimental designs such as those used in the current protocol cannot be generalized beyond the participants included in these specific trials. However, in samples of disorders characterized by high heterogeneity amongst individuals, such as observed in adults with DD (Gregg et al., 2006; Swanson, 2012), single-subject designs may better illustrate treatment outcomes (Tate et al., 2013; Krasny-Pacini and Evans, 2018). In this study, we follow SCRIBE guidelines (Tate et al., 2016) for reporting single-subject outcomes in an effort to highlight the importance of single-subject data because group average analyses may risk removing unique sources of intersubject variability in treatment outcomes. This is particularly important in this case, not only due to the heterogeneity observed in DD but also due to the high levels of variability in outcome response associated with neuromodulation (Nicolo et al., 2015; Corp et al., 2020). Future research should include large scale randomized, controlled trials with a large enough sample to address these limitations.

Despite the strength of the single-subject design for representing treatment effects, the current study suffered from participant heterogeneity and protocol deviations which can reduce internal validity. Specifically, participants with comorbid disorders such as ADHD and depression/anxiety were not excluded from the study if the disorder was well controlled and were not taking medications that were contraindications to TBS. While this represents the high rate of comorbidity often observed in the DD population (Moll et al., 2020), it may serve as a confound factor in treatment outcomes. With respect to protocol deviations, 13 out of 14 participants completed all 20 treatment sessions, with one participant being disqualified after 8 sessions due to excessive absences. Three cases required a change in protocol such that one received instruction only, one received baseline only (sham + instruction) and one was switched to instruction only after 8 baseline and 7 experimental sessions. Although unintended, these protocol deviations allowed for comparison cases to help assess the effect of instruction only learning effects.

It is also important to note that the findings presented in the current manuscript are presented for visualization purposes only and do not represent direct statistical analyses. This is of particular importance with respect to the fMRI findings. Traditional neuroimaging analyses are best used with large samples where group or pre/post treatment comparisons can be performed. The current small sample and single-subject design are prohibitive to this approach. As such, the presented representative visualizations have a liberal threshold (*p* < 0.01 uncorrected) with an increased risk of a high false positive rate. In an effort to address this, we present cases that represent similar trends noted across all (or most) participants. We also present findings from two comparison cases that do not demonstrate the same trends. This type of visualization for pre-post treatment effects within-subject has been previously used in single-subject rTMS treatment studies (Martin et al., 2009) and is a useful way to represent neural changes in high and low responders, despite lacking statistical validity. Previous studies have also used single-subject fMRI to represent neural differences and treatment gains in atypical populations (Turkeltaub et al., 2004; Rojo et al., 2011; Schuster-Amft et al., 2015; Gabbatore et al., 2017). Within-subject analysis is particularly relevant in adults with poor reading skills because high interindividual variability in neural activity (likely due to individual compensatory strategies during development as well as genetic predisposition) may ultimately obscure true treatment-related changes when analyzed at the group level (Centanni et al., 2018).

Participants in this study received SRA intervention in combination with aTBS. This presents a significant limitation in that our results cannot be attributed to aTBS stimulation alone. We attempted to address this limitation in several ways. First, two control cases were included in the study, one who received sham stimulation with SRA instruction and one who participated only in SRA instruction. These two cases differed from participants who received active combined stimulation and SRA in a number of ways post-treatment as described above. Additionally, we detrended the data using an approach designed to remove ongoing behavioral effects (Solanas et al., 2010). This approach removes the trend from the baseline phase from the data for the treatment phase to remove the expected improvements seen from instruction alone. While several approaches are available to remove the effect of a pre-condition, choosing an approach that focuses on slope and trend removal is particularly important in a learning-based experimental design (Kratochwill et al., 2023).

Despite methodological limitations of this design, combined aTBS treatment with an established standard of care treatment like SRA was a purposeful, principle-driven decision. This study required a significant time commitment from participants with poor reading abilities. This population faces frequent educational and socioeconomic barriers which motivated a design in which all participants received a validated treatment regardless of their study assignment. Although instruction alone was not hypothesized to produce meaning behavioral improvements, delivering SRA intervention alongside the novel aTBS intervention ensured participants received a well validated reading intervention while the novel approach was also evaluated (Hart and Bagiella, 2012). This layering approach is frequently used in clinic trials when withdrawal of standard of care would be considered unethical (Dromerick et al., 2021; Gillam et al., 2022). While our participants were not subject to withdrawal of care, we considered the substantial time commitment and the vulnerability of the population warranted a similar approach. Finally, brain stimulation with behaviorally relevant training has been shown more effective than stimulation alone (Koganemaru et al., 2015). SRA is an evidence-based reading intervention that targets decoding, fluency, and oral reading accuracy at an individual level. We believed that highly structured instruction immediately following stimulation would further engage the targeted networks. Neuroplasticity is activity-dependent and this design aims to capitalize on that by pairing stimulation with evidence-based intervention (Kleim and Jones, 2008).

Additionally, although the accelerated treatment design reduced the required number of treatment days, it produced additional confounding limitations. The extended time commitment associated with study participation presented challenges for participant recruitment. For instance, three potential participants that qualified into the treatment portion of the study were unable to participate due to conflicts related to school or work obligations. During treatment, the extended hours in addition to accelerated treatment demands led to participant fatigue, observed in both the experimental and comparison participants. This may have influenced the quality or consistency of behavioral data collected during the afternoon sessions. Specifically, morning to afternoon variability was observed in several participant’s behavioral responses (e.g., performance accuracy, reaction times). This inherent variability, especially when aggregated, impacted the effectiveness of data detrending techniques designed to isolate the treatment effect, potentially obscuring subtle but significant changes.

Future research should aim to refine data processing protocols to more effectively handle inherent behavioral variability and consider implementing shorter, more frequent treatment sessions to mitigate participant fatigue. Most importantly, while the acute behavioral effects of the intervention were observed, the current study could not assess sustained outcomes; therefore, a critical future direction is to design longitudinal studies to evaluate the long-term effects and durability of the observed behavioral changes, providing a more complete understanding of its real-world impact and clinical utility. This multiple baseline design with randomized phase transition allows for the assessment of the unique contribution of aTBS to treatment outcomes. Overall, the current study design provides promising preliminary evidence for the use of combined brain stimulation and behavioral instruction to improve reading outcomes for treatment-resistant, persistent DD.

## Data Availability

The datasets presented in this study can be found in online repositories. The names of the repository/repositories and accession number(s) can be found at: Collaborative Informatics and Neuroimaging Suite Data Exchange at https://coins.trendscenter.org/.
