# Supplementary figures and images for: Combined accelerated theta burst stimulation and reading instruction for the treatment of persistent developmental dyslexia: methodology and preliminary findings
Source: Front Hum Neurosci. 2026 Apr 24;20:1758532. doi: 10.3389/fnhum.2026.1758532 (PMC13153044; doi:10.3389/fnhum.2026.1758532)

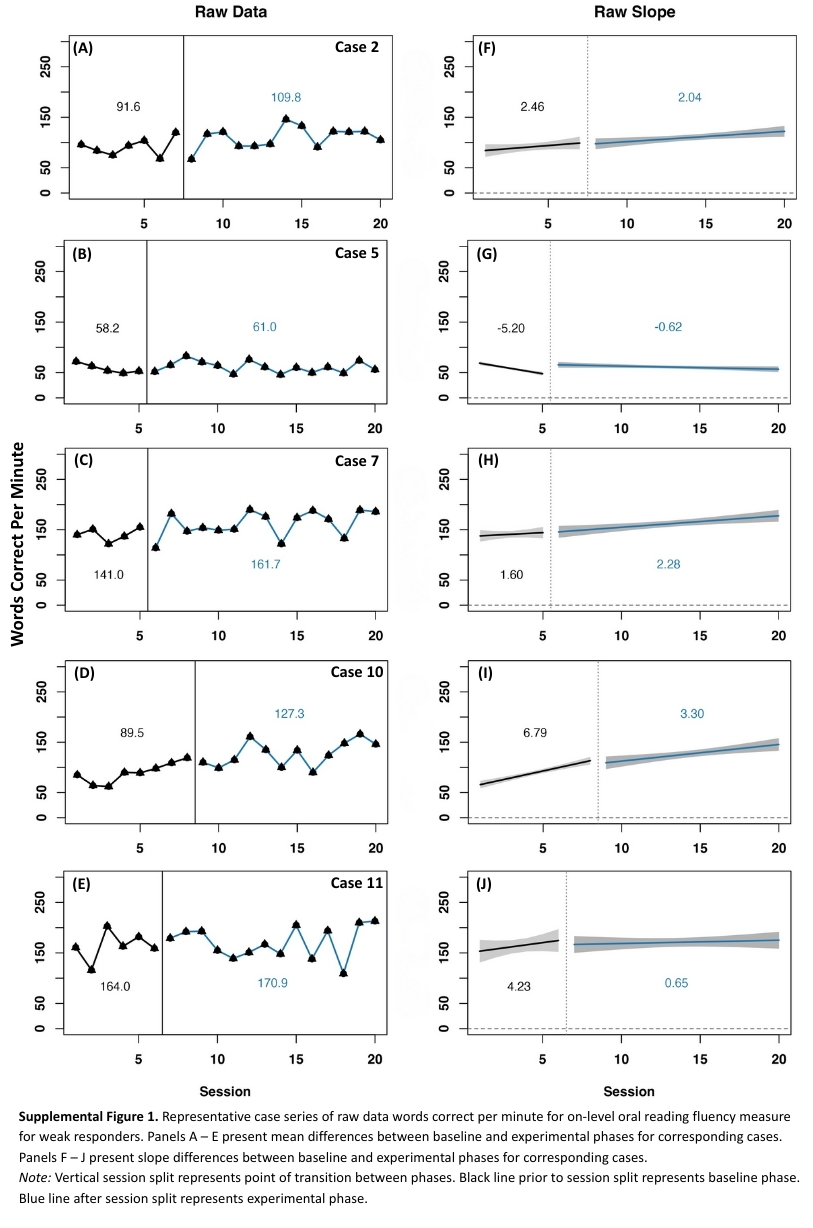

Supplement: Supplementary file 1 [file Data_Sheet_1.ZIP › Supplementary materials presentation/Sup1_OL.jpg]

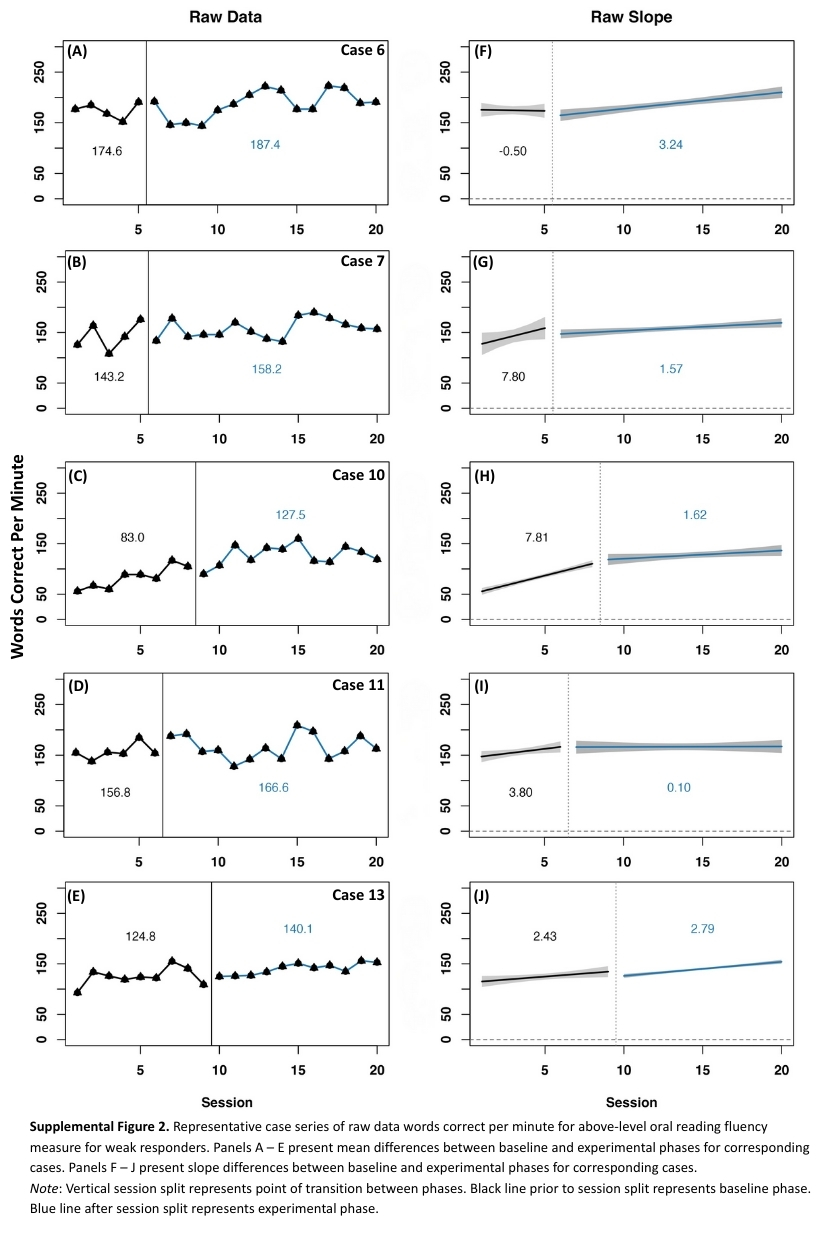

Supplement: Supplementary file 1 [file Data_Sheet_1.ZIP › Supplementary materials presentation/Sup2_AL.jpg]

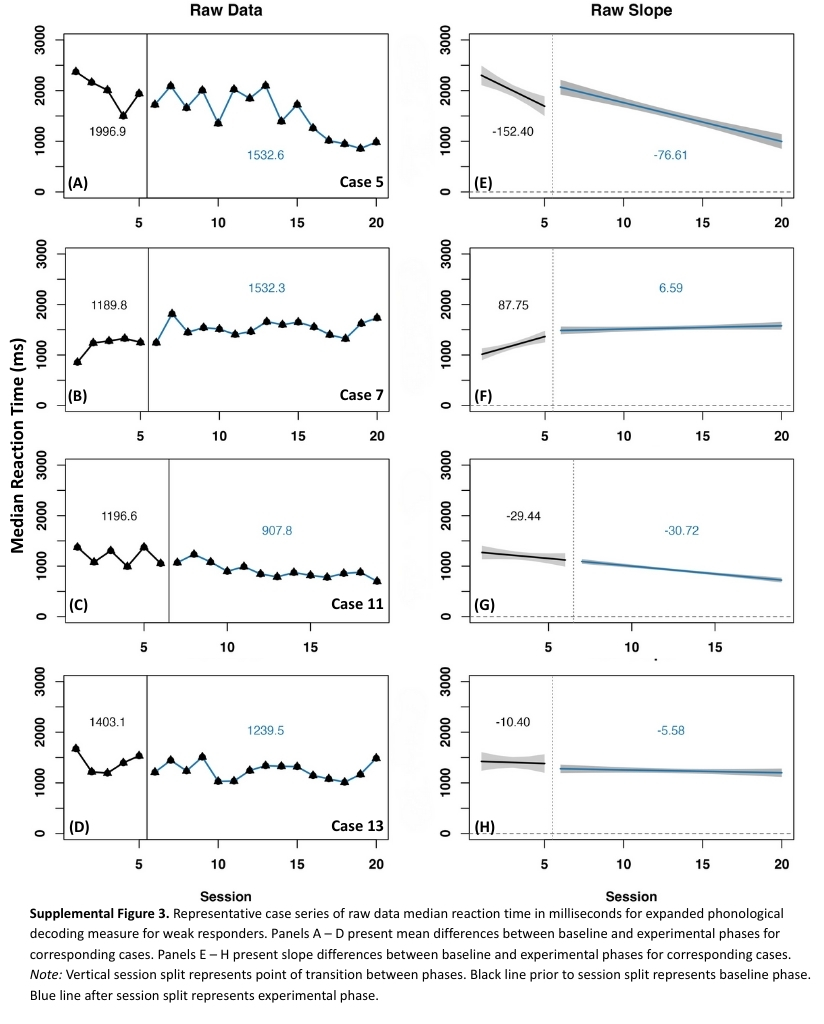

Supplement: Supplementary file 1 [file Data_Sheet_1.ZIP › Supplementary materials presentation/Sup3_PD.jpg]

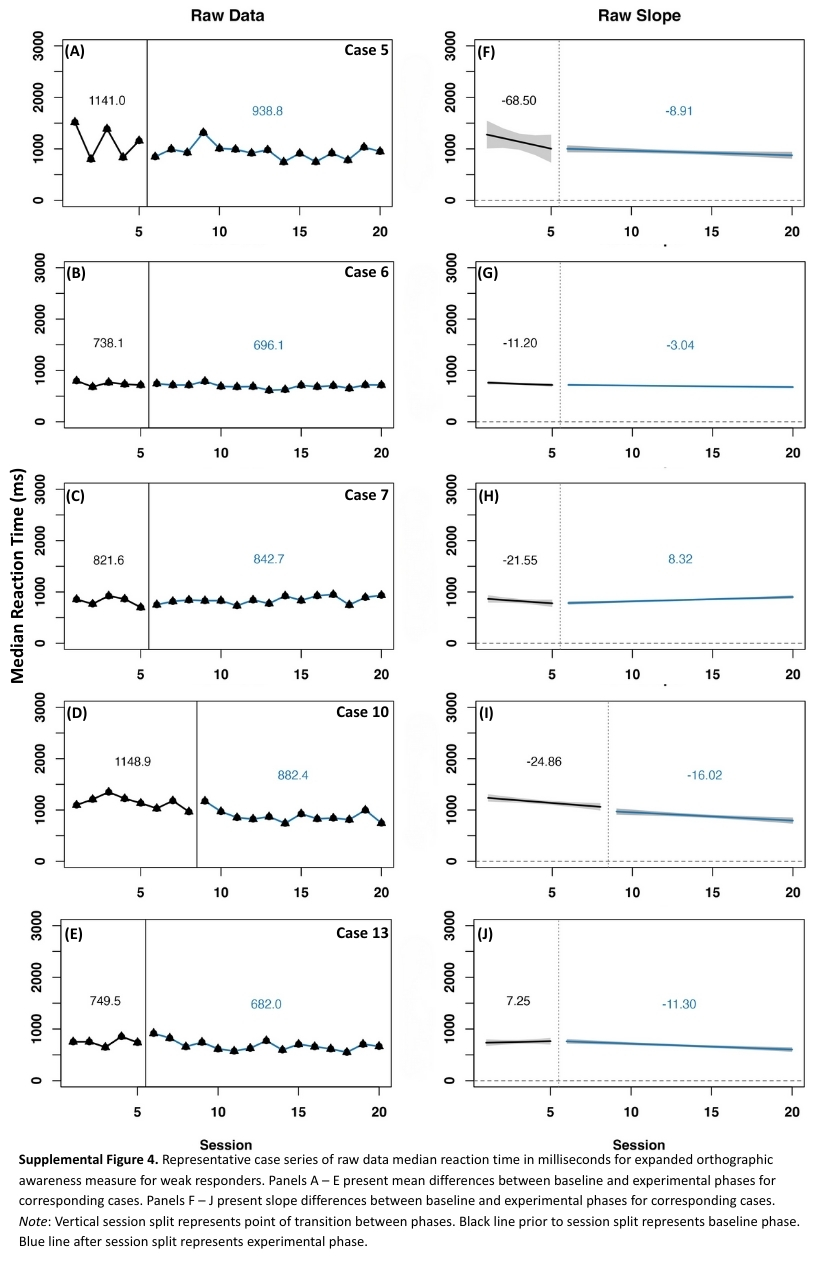

Supplement: Supplementary file 1 [file Data_Sheet_1.ZIP › Supplementary materials presentation/Sup4_OA.jpg]
